# Activation of Wnt signalling reduces the population of cancer stem cells in ameloblastoma

**DOI:** 10.1111/cpr.13073

**Published:** 2021-06-06

**Authors:** Hyun‐Yi Kim, Shujin Li, Dong‐Joon Lee, Jin Hoo Park, Takashi Muramatsu, Hidemitsu Harada, Young‐Soo Jung, Han‐Sung Jung

**Affiliations:** ^1^ Division in Anatomy and Developmental Biology, Department of Oral Biology, Oral Science Research Center, BK21 FOUR Project Yonsei University College of Dentistry Seoul Korea; ^2^ Department of Oral & Maxillofacial Surgery Yonsei University, College of Dentistry Seoul Korea; ^3^ Department of Operative Dentistry, Cariology and Pulp Biology Tokyo Dental College Tokyo Japan; ^4^ Division of Developmental Biology and Regenerative Medicine, Department of Anatomy Iwate Medical University Iwate Japan

**Keywords:** ameloblastoma, cancer stem cell, Wnt signalling

## Abstract

**Objectives:**

The treatment of ameloblastoma, an odontogenic epithelial tumour destroying jawbone, mainly depends on radical destructive resections. Other therapeutic options are limited by the characteristics of ameloblastoma, such as high recurrence rates and resistance to radiation and chemotherapy, which implies possible existence of cancer stem cells (CSCs) in ameloblastoma. Here, we identified a putative CSC population in immortalized and primary human ameloblastoma cells and examined possible therapeutic reagents to reduce the CSC population.

**Methods:**

We investigated subpopulations of AM**‐**1 cell line and human ameloblastoma cells using immunocytochemistry and flow cytometry and the effects of Wnt signalling activators on the 2**‐** and 3**‐**dimensional cultured ameloblastoma cells using molecular biological analyses.

**Result:**

Among heterogenous ameloblastoma cells, small**‐**sized and round**‐**shaped cells were found to be proliferative and expressed a marker of dental epithelial stem cells, SRY**‐**box 2 (Sox2). Exogenous activation of Wnt signalling using glycogen synthase kinase 3β inhibitors, lithium chloride (LiCl) and valproic acid (VPA), increased the cell size and decreased proliferation of cells and expression of Sox2 in 2 dimensionally cultured AM**‐**1 and human primary ameloblastoma cells. Furthermore, the growth of 3 dimensionally cultured AM**‐**1 cells as suspended or embedded in gel was suppressed by treatment with Wnt signalling activators, VPA and CHIR99021, or antibodies to sclerostin, an antagonist of Wnt signalling.

**Conclusion:**

We suggest that Wnt signalling activators are potential drug candidates to suppress CSCs in ameloblastoma.

## Graphical abstract

Ameloblastoma, a benign tumour of the odontogenic epithelium, presents significant recurrence rates and resistance to radiotherapy and chemotherapy, which compel patients to undergo radical resections, potentially causing surgical morbidity. In this study, we found that the exogenous activation of Wnt signalling using antibody (α**‐**SOST antibody) or chemicals (LiCl, VPA or CHIR9902) increases β**‐**catenin (β**‐**cat) in ameloblastoma, leading to an increase in β**‐**cat^high^ and Sox2**^‐^** cells and decrease in β**‐**cat^low^ and Sox2^+^ cells, the putative cancer stem cells (CSCs). This change of sub**‐**population in ameloblastoma results in the suppression of spheroid**‐**forming activity and invasiveness. The results suggest that Wnt signalling activators may serve as potential drug candidates to suppress CSCs in ameloblastoma.

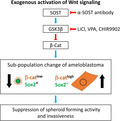

## 1 INTRODUCTION

The cancer stem cell (CSC) model is a prominent concept to explain heterogeneity of tumours.^1^ CSCs have been revealed to be a self**‐**renewing subpopulation in tumours that generate various differentiated cell populations. Characterization of CSCs has indicated that they are remarkably resistant to conventional radiotherapy and chemotherapy. Clinically, the residual populations of CSCs are responsible for metastasis and recurrence in cancer patients, which can lead to chronic and incurable cancers. Therefore, elimination of CSCs is an important goal for treatment of cancer.^2^

Ameloblastoma is the most common odontogenic tumour, accounting for 1% of all tumours of the head and neck region and around 11% of all odontogenic tumours. Classification of ameloblastomas has currently been established, which includes three types**‐**conventional, unicystic and extraosseous/peripheral.^3^ Its biological behaviour is considered to be more aggressive due to its higher incidence of recurrence ^4^ ; consequently, the current standard treatment is wide resection with appropriate margins and immediate reconstruction, which is associated with significant patient morbidity.^5^ Although radiotherapy has been attempted for decreasing the recurrence rate, its efficacy is not clear.^5^ A systemic genomic analysis showed that over 80% of ameloblastomas harbour oncogenic mutations in the sonic hedgehog (SHH) and mitogen**‐**activated protein kinase (MAPK) pathways.^6^

AM**‐**1 is an ameloblastoma cell line that is immortalized using human papillomavirus type**‐**16.^7^ The AM**‐**1 cell line exhibits characteristics that are similar to in situ ameloblastoma cells related to marker expression and invasive properties, which shows that this cell line is an appropriate model system to study ameloblastoma.^7^, ^8^, ^9^, ^10^ Studies using this cell line revealed that Akt, MAPK and SHH signalling pathways are related to proliferation and apoptosis of AM**‐**1.^11^, ^12^, ^13^, ^14^, ^15^ Interestingly, the Wnt pathway, an osteogenic signalling pathway, is suppressed in this cell line.^16^, ^17^ Early progeny of Sox2**‐**positive dental epithelial stem cells (DESCs) are known to transiently express a Wnt inhibitor, secreted frizzled**‐**related protein (Sfrp) 5.^18^ AM**‐**1 cells also express the Wnt antagonist Sfrp2,^16^ and osteogenic genes related to Wnt signalling are suppressed in this cell line.^17^ A recent study showed that Wnt signalling is important for enamel formation by facilitating ameloblast differentiation and movement.^19^

In this study, we demonstrated the presence of a putative CSC population in AM**‐**1, a well**‐**established human ameloblastoma cell line. Immunocytochemistry and flow cytometry of AM**‐**1 cells showed that small**‐**sized and round**‐**shaped cells were proliferative and expressed a marker of DESCs, SRY**‐**related HMG box 2 (Sox2).^18^, ^20^, ^21^ Interestingly, Sox2 expression in the cells was negatively correlated with activation of Wnt signalling. We examined the effect of various exogenous Wnt activators in 2 dimensionally (2D) or 3 dimensionally (3D) cultured AM**‐**1 cells and human primary ameloblastoma cells. These Wnt activators showed an inhibitory effect in growth of ameloblastoma cells.

## 2 METHODS

### 2.1 Cell culture, 3‐dimensional culture and cell sheet generation

AM**‐**1 cells were cultured in keratinocyte growth medium supplemented with pituitary extract (Gibco, Grand Island, NY, 17005**‐**042) at 37°C and 5% CO_2_ in a humidified incubator. For spheroid formation, 1.0 × 10^5^ or 2 × 10^5^ cells were plated onto Ultra**‐**Low Attachment Surface Costar 6 Well Plates (Corning Inc, Corning, NY, 4371) with keratinocyte growth medium or Dulbecco's Modified Eagle's Medium (DMEM, Gibco, 11995**‐**065) supplemented with 10% foetal bovine serum (FBS, Gibco, 12484**‐**020) and 1% penicillin/streptomycin (Gibco, 15140**‐**112) solution at 37°C for 1 week. For 3**‐**dimensional culture in gels, 1.0 × 10^3^ cells were suspended in 40 μl of gels and spot into one well of the pre**‐**warmed 24 well plate. The gels were solidified in 37°C incubator and then keratinocyte growth media supplemented with 0, 0.6 or 1.2 mmol/L calcium chloride. For cell sheet generation, AM**‐**1 cells were plated onto temperature**‐**responsive dishes (Nunc UpCell 3.5 cm dish, ThermoFisher Scientific, Somerset, NJ, NUN**‐**174904) and cultured with keratinocyte growth medium until fully confluent. The confluent cells were detached in the form of a cell sheet as described in the manufacturer's instructions.

### 2.2 **Real**‐**time PCR analysis**

Total RNA of AM**‐**1 cells cultured with conventional cell culture methods or cultured as spheroids were extracted using Trizol™ reagent (Invitrogen Corp. 16596026) as described in the manufacturer's instructions. The extracts were reverse**‐**transcribed using Maxime RT PreMix (iNtRON, 25081). The products were subjected to real**‐**time PCR analyses with primer sets (*Oct3/4*, F 5'**‐**CTG GGC TCT CCC ATG CAT**‐**3', R 5'**‐**CCT GTC CCC CAT TCC TAG AAG**‐**3'; *Sox2*, F 5'**‐**ACA GCA AAT GAC AGC TGC AAA**‐**3', R 5'**‐**TCG GCA TCG CGG TTT TT**‐**3'; *CD49f*, F 5'**‐**GAT CCC GGC CTG TGA TTA ATA TT**‐**3', R 5'**‐**CTG GCG GAG GTC AAT TCT GT**‐**3'; *Bcl11b*, F 5'**‐**GCT GGG TCC AGG TGA AGT GA**‐**3', R 5'**‐**CGA AAG GTC CTG GCT GTG AT**‐**3'; *Axin2*, F 5'**‐**CCA AGC AGA CGA CGA AGC AT**‐**3', R 5'**‐**GTT TCC GGA GCC TTG GAG TG**‐**3'; *GAPDH*, F 5'**‐**GAA GGT GAA GGT CGG AGT**‐**3', R 5'**‐**GAA GAT GGT GAT GGG ATT TC**‐**3') using the StepOnePlus Real**‐**Time PCR System (ThermoFisher Scientific).

### 2.3 Immunoblot analysis

AM**‐**1 cells were lysed in protein extraction buffer with a protease inhibitor cocktail (cOmplete Mini, Roche, 11836170001). The lysates were subjected to immunoblot analyses using anti**‐**β**‐**catenin (Santa Cruz Biotechnology, Santa Cruz, CA, sc**‐**7199) and anti**‐**α**‐**tubulin (Sigma**‐**Aldrich, T6199). For visualization, anti**‐**mouse or rabbit IgG conjugated with horseradish peroxidase (Santa Cruz Biotechnology, sc**‐**2005 and sc**‐**2004, respectively) was applied and visualized with ECL (GE Healthcare, RPN2232) using a chemiluminescence imaging system (Davinch**‐**chemi, Core Bio).

### 2.4 Flow cytometry

AM**‐**1 cells detached using trypsin**‐**EDTA were centrifuged and resuspended in keratinocyte growth medium. Light scattering characteristics of cells were analysed using forward scattered light (FSC) and side**‐**scattered light (SSC), and based on the characteristics, cells were sorted into two subpopulations using a BD FACSAria III cell sorter (BD Biosciences).

### 2.5 Animal experiments

Female nude (nu/nu BALB/c/Bkl) mice (Nara Biotech Co.) were housed in a temperature**‐**controlled room (22°C) under artificial illumination (lights on from 05:00 to 17:00) and 55% relative humidity. Mice had access to food and water ad libitum. For orthotopic grafts, the upper first molars of 8**‐**week**‐**old nude mice were extracted, and a hole was prepared using a portable drill with a 0.75 mm tip in the extraction site under deep anaesthesia. Subsequently, a properly sized AM**‐**1 cell sheet was grafted into the hole using forceps. AM**‐**1 cell sheet**‐**grafted mice were housed for 1 week for healing and subsequently sacrificed with CO_2_ for histological and immunohistological analyses.

### 2.6 Immunocytochemical and immunohistochemical analysis

Cells were fixed in 4% paraformaldehyde (PFA) and permeabilized with 0.02% Triton X**‐**100 in phosphate**‐**buffered saline. Spheroids or decalcified tissues were fixed in 4% PFA. Staining was performed on 4 μm paraffin**‐**embedded sections. After deparaffinization, the slides were incubated with pepsin (Digest**‐**All™ 00**‐**3009, Invitrogen) for 15 minutes at 37°C. After blocking with 5% bovine serum albumin, cells were incubated with the following primary antibodies: anti**‐**β**‐**catenin (Santa Cruz Biotechnology, sc**‐**7199), anti**‐**Sox2 (Santa Cruz Biotechnology, sc**‐**20088) and anti**‐**Ki67 (Spring Bioscience Corp., M3060). For visualization, anti**‐**mouse or rabbit IgG conjugated with Alexa Fluor 488 or 555 dye (Invitrogen) was applied and observed under a confocal microscope (LSM700, Carl Zeiss,). The cytoskeleton or nucleus was stained using phalloidin conjugated with Alexa Fluor 488 dye (Invitrogen) or 4',6**‐**diamidino**‐**2**‐**phenylindole (ThemoFisher Scientific, D1306), respectively. The cell size and staining intensity were measured using the image analyser software ImageJ 1.51 g (National Institutes of Health, Bethesda, MD).

## 3 RESULTS

### 3.1 **A putative CSC population in AM**‐**1 cells**

**FIGURE 1:**
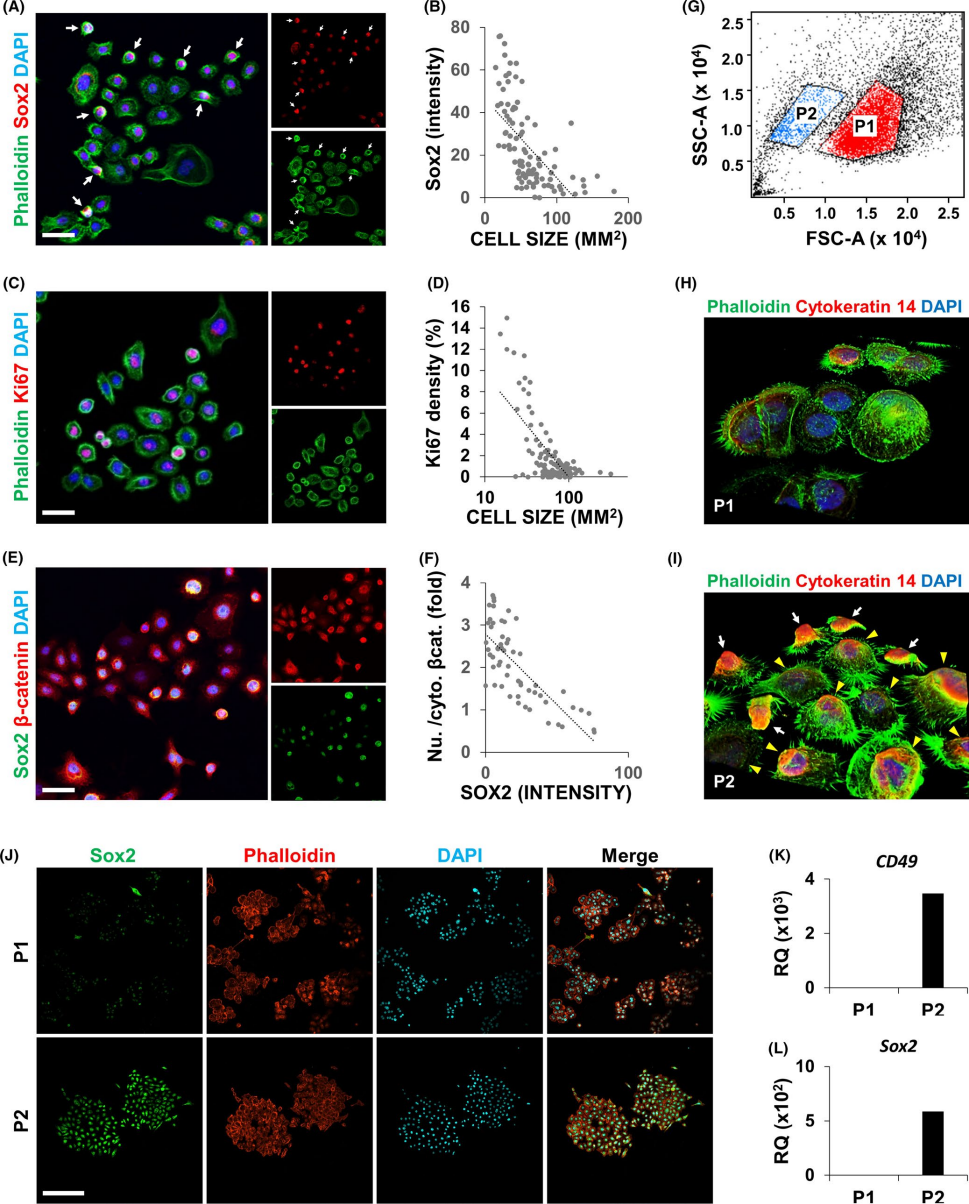
AM‐1 harbours a cancer stem cell‐like population. A**‐**F, AM**‐**1 cells were subjected to immunocytochemistry (ICC) using antibodies to Sox2 (A, E), Ki67 (B), or β**‐**catenin (E). Small round cells strongly expressing Sox2 are indicated by white arrows (A). The cell size (B, D), Sox2 intensity (B, F), or ratio of nuclear to cytosolic β**‐**catenin (F) of each cell were measured and are displayed as a dot plot. A trend line (dotted line) shows the relationship between the measured factors (B, D and F). Cytoskeletons (A and C) and nuclei (A, C and E) were visualized using Alexa 488**‐**conjugated phalloidin (green) and DAPI (blue), respectively. Scale bar = 20 μm. G**‐**J, Scattered light characteristics of AM**‐**1 cells based on forward scattered light (FSC) and side scattered light (SSC). AM**‐**1 cells were sorted into two groups (P1 and P2) based on FSC and SSC (G) and cultured for 1 (H, I) or 3 d (J). Three dimensionally reconstructed image (H, I) and conventional confocal image (J). The cells were subjected to ICC using an antibody to Cytokeratin 14 and Sox2 (H**‐**J). Cytoskeletons and nuclei (H**‐**J) were visualized using fluorophore**‐**conjugated phalloidin and DAPI (blue), respectively. Scale bar = 100 μm. K**‐**L, Total RNA of AM**‐**1 cells in P1 and P2 were extracted and subjected to real**‐**time PCR analyses using primer sets of *CD49f* (K) and *Sox2* (L). RQ, relative quantity. n = 3

As previously described,^7^ AM**‐**1 cells exhibited a heterogeneous morphology**‐**from small round cells to large flattened cells. The average cell size was 60.6 ± 32.4 μm^2^, and the largest cell was 12 times larger than the smallest one. To detect a possible CSC population inside these heterogeneous cells, Sox2, a marker of DESCs,^18^, ^20^, ^21^ was stained and visualized (Figure 1A, red). Sox2 expression significantly differed among the cells and was especially strong in small round cells (Figure 1A, arrows). Quantification of the results revealed a negative correlation between Sox2 expression and the cell size of AM**‐**1 cells (Figure 1B, Pearson's correlation coefficient r = −0.616). Additionally, the expression of Ki67, a marker of cell proliferation, showed a wide variation among the cells (Figure 1C) and was also negatively correlated with cell size in AM**‐**1 cells (Figure 1D r = −0.691).

Wnt signalling, an essential signalling pathway for ameloblast differentiation, is known to be suppressed in DESCs and AM**‐**1 cells.^17^, ^18^, ^19^ Staining of β**‐**catenin, an effector molecule of Wnt signalling, revealed that it was localized differently in AM**‐**1 cells; some cells displayed the presence of nuclear β**‐**catenin, which indicates activation of Wnt signalling, but cytosolic β**‐**catenin was observed in other cells (Figure 1E). Quantitative analysis showed that the nuclear localization of β**‐**catenin was negatively correlated with Sox2 expression (Figure 1F r = −0.734). Overall, small round cells showed significant proliferation as well as a high expression of stem cell markers with low Wnt signalling activity, which identified them as putative CSCs in AM**‐**1 cells.

Flow cytometry analysis using FSC and SSC displayed two subpopulations of cells with different sizes in AM**‐**1 cells (Figure 1G). After size**‐**based cell sorting using flow cytometry, we separately cultured each population for one day (Figure S1 and Figure**** 1H‐L). Cells expressing cytokeratin 14, a marker of epithelial stem cells, were found in P2 (Figure 1H, white arrows), which were relatively small compared with cytokeratin 14**‐**negative cells (Figure 1I, yellow arrowheads). Cells in both the populations formed larger colonies after a 3**‐ ‐**culture, and the expression of Sox2 was much higher in the colonies of P2 compared with those of P1 (Figure 1J). Real**‐**time PCR analysis showed enrichment of stemness markers (*CD49* and *Sox2*) in the cells of P2 (Figure 1K,L).

### 3.2 **Effect of Wnt signalling activators on putative CSC population in AM**‐**1 cells**

**FIGURE 2:**
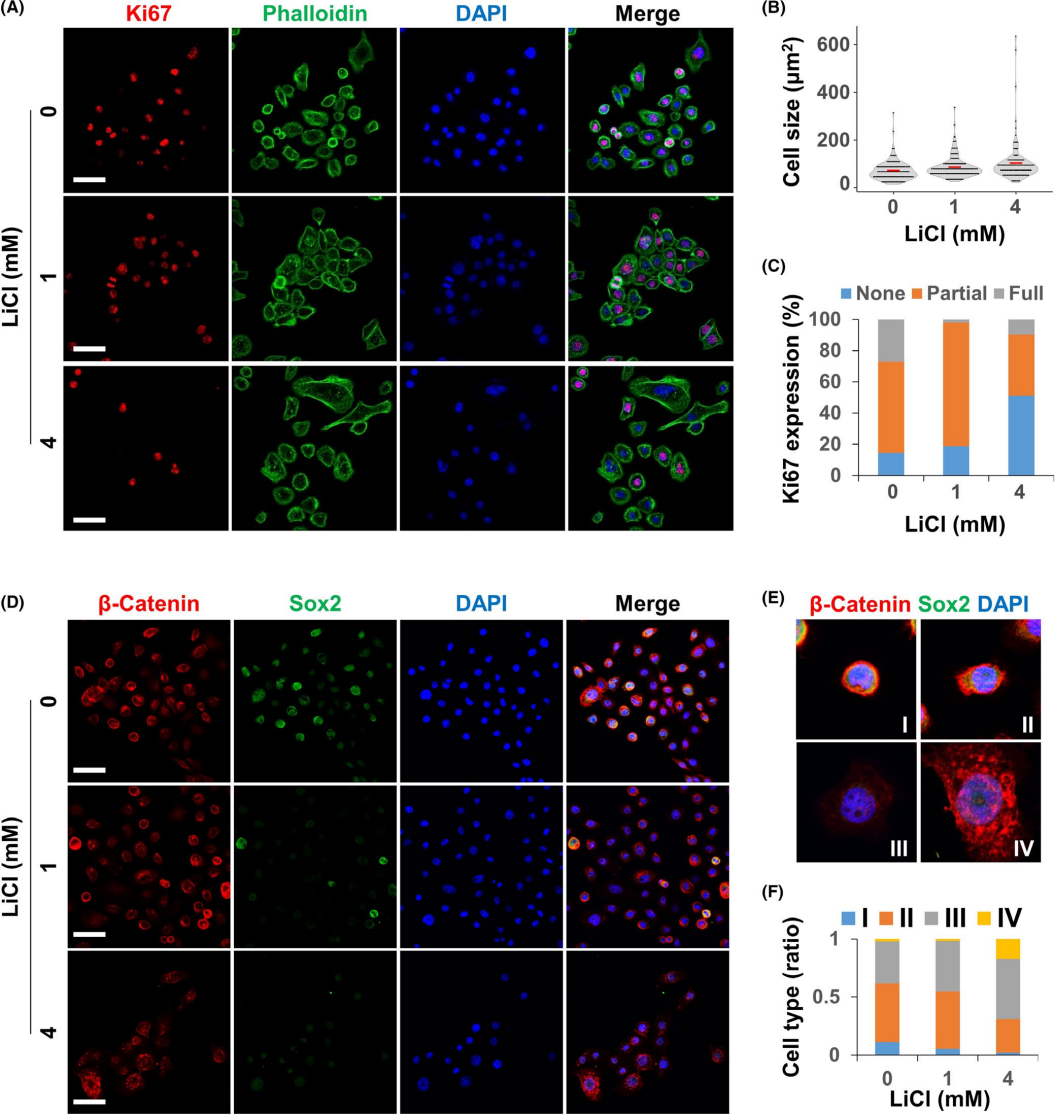
Treatment with lithium chloride increased the cell size and decreased proliferation and expression of stem cell markers of AM**‐**1 cells. A**‐**F, AM**‐**1 cells were cultured for 24 h with the indicated dose of lithium chloride (LiCl). The cells were subjected to ICC using antibodies to Ki67 (A), β**‐**catenin, or Sox2 (D). Cytoskeletons (A) and nuclei (A, D) were visualized using Alexa 488**‐**conjugated phalloidin (green) and DAPI (blue), respectively. Distribution of cell size (B) and ratio of cell populations classified by Ki67 expression pattern (C; none, partially, or fully covered nucleus with Ki67) or by size, shape, Sox2 expression and nuclear accumulation of β**‐**catenin (E and F, class I**‐**IV) were analysed and are displayed as a graph (B, C and F). The average cell size in each group is indicated by a red bar (B). Scale bar = 20 μm

**FIGURE 3:**
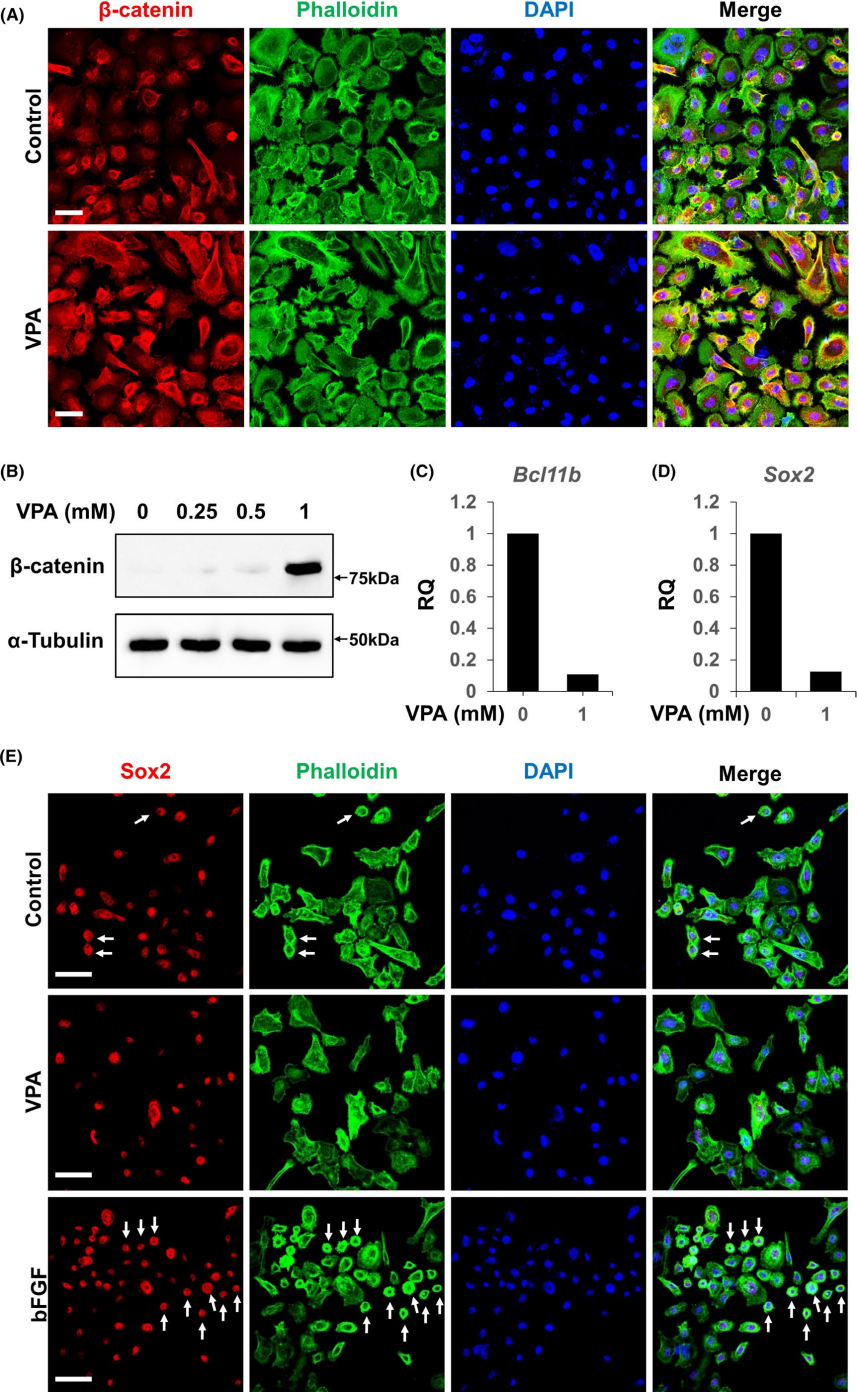
Treatment with valproic acid increased the cell size and decreased proliferation and expression of stem cell markers of AM**‐**1 cells. A**‐**E, AM**‐**1 cells were cultured for 24 h with 1 mmol/L valproic acid or 100 mg/ml basic fibroblast growth factor (bFGF). The cells were subjected to ICC using antibodies to β**‐**catenin (A) and Sox2 (E), and immunoblotting using antibodies to β**‐**catenin or α**‐**tubulin (B). Cytoskeletons and nuclei were visualized using Alexa 488**‐**conjugated phalloidin (green) and DAPI (blue), respectively (A, E). Scale bar = 20 μm. Total RNA of the cells was extracted and subjected to real**‐**time PCR analyses using primer sets of *Bcl11b* (C) and *Sox2* (D). n = 3. VPA, valproic acid. RQ, relative quantity

To further investigate the role of Wnt signalling in putative CSCs, we activated Wnt signalling in AM**‐**1 cells by using the Wnt signalling activators lithium chloride (LiCl) and valproic acid (VPA) to assess cellular and molecular changes (Figures 2,3). Upon treatment with LiCl, the number of large flattened cells without Ki67 expression increased (Figure 2A‐C). Sox2 also showed a dose**‐**dependent decrease after the LiCl treatment (Figure 2D). We classified AM**‐**1 cells into four types (Figure 2E) according to size (small or large), shape (round or flat), Sox2 expression (Sox2^high^ or Sox2^low^) and nuclear accumulation of β**‐**catenin (nuclear β**‐**cat^high^ or nuclear β**‐**cat^low^). Upon treatment with LiCl, type I (small, round, Sox2^high^ and nuclear β**‐**cat^low^) and type II (small, dented, round, Sox2^high^ and nuclear β**‐**cat^low^) cells increased, whereas type III (small, flat, Sox2^low^ and nuclear β**‐**cat^low^) and type IV cells (large, flat, Sox2^low^ and nuclear β**‐**cat^high^) decreased (Figure 2F).

Treatment with VPA showed a similar result as that of LiCl. The number of large flat cells and expression of β**‐**catenin in the cells increased (Figure 3A,B). The transcription of Axin2, a target gene of Wnt signalling, also increased in dose**‐**dependent manner by VPA treatment (Figure S2). Expression of *Bcl11b* and *Sox2* decreased upon VPA treatment (Figure 3C,D). An opposite effect was observed in cells treated with basic fibroblast growth factor (bFGF), a mitogenic factor that stimulates ameloblastoma proliferation ^15^ ; the number of small, round, Sox2^high^ cells increased upon bFGF treatment (Figure 3E, arrows). These results show that small, round, Sox2^high^ cells respond to their surrounding microenvironment, which is one of the basic features of CSCs.^2^

### 3.3 **In vitro spheroid**‐**forming capacity of AM**‐**1 cells**

In vitro spheroid**‐**forming assay is a well**‐**established method for demonstrating self**‐**renewal capacity of stem cells from various organs.^22^ We plated various numbers of AM**‐**1 cells on low attachment surface cell culture plates with several different culture media to optimize spheroid**‐**forming conditions (Figure S3A). No spheroid formation was observed when the seeded number of cells was 2 × 10^5^ per well in a 6‐well plate with keratinocyte growth medium (Figure S3A). However, the same number of cells grown in DMEM displayed spheroid formation (Figure S3A). Interestingly, the dissected spheroids showed a similar structure to that of ameloblastoma; the hyperchromatic outer shell implied the presence of peripheral palisading cells at the basal layer of ameloblastoma, and the eosinophilic spots inside the spheroids were similar to keratin pearls, typical structures found in acanthomatous ameloblastoma (Figure S3B). Real**‐**time PCR showed that the expression of stem cell markers (*OCT3/4*, *Sox2* and *CD49f*) and an anti**‐**apoptotic marker (*Bcl11b*) increased in cells that were three dimensionally (3D) cultured as spheroids compared with those cultured in a conventional cell culture system (Figure S3C‐F).

### 3.4 In vivo tumour‐forming capacity of AM‐1 cells

In vivo tumour**‐**forming capacity of AM**‐**1 cells was assessed by orthotopic grafts of ameloblastoma cells (Figure S4A‐D). A mass of AM**‐**1 cell sheet was implanted into a hole drilled at the extraction site of the maxillary first molar of 8 week**‐**old BALB/c nude mice (Figure S4A,B). After a week, complete closure of the extraction site was observed (Figure S4B, the area surrounded by the black dotted line). Histological analysis of the maxillary tissue revealed the formation of an abnormal cell mass at the extraction site (Figure S4C, upper panel). Immunostaining for a human**‐**specific antigen, human leukocyte antigen (HLA), showed the existence of exogenous cells in the mass (Figure S4C, lower panel). The exogenous cells formed eosinophilic structures that were suggestive of those observed inside AM**‐**1 spheroids (Figure S4D, arrows).

### 3.5 Effect of Wnt signalling activators on human primary ameloblastoma cells

**FIGURE 4:**
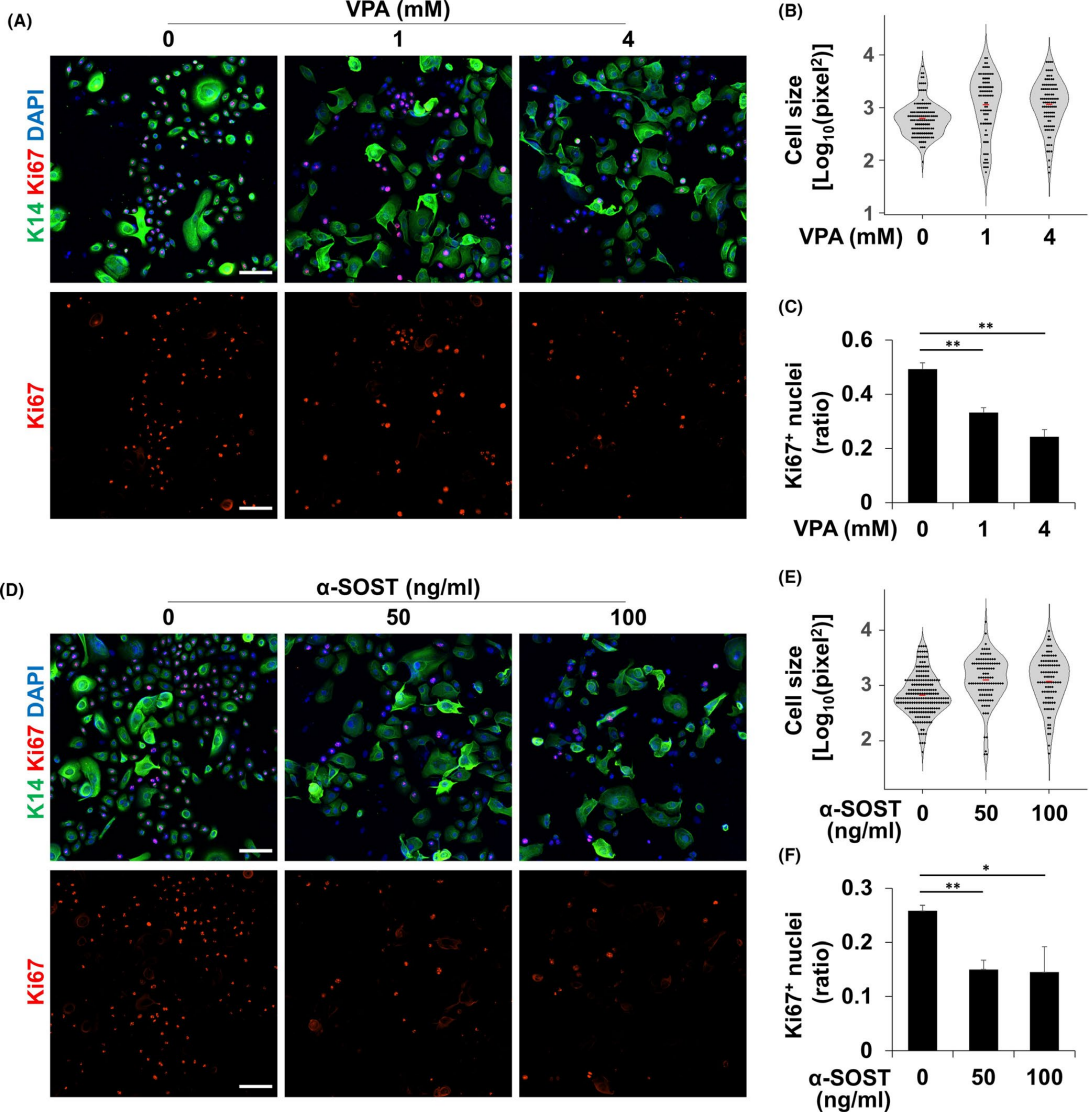
Treatment with VPA or α**‐**SOST increased the cell size and decreased proliferation and expression of stem cell markers of AM**‐**1 cells. A**‐**F, AM**‐**1 cells were cultured for 24 h with the indicated dose of VPA or α**‐**SOST. The cells were subjected to ICC using antibodies to cytokeratin 14 (K14, green) and Ki67 (red). Nuclei were visualized using DAPI (blue), respectively. Cell size (B and E) and ratio of Ki67**‐**positive cells per total cells (C and F) were analysed and are displayed as a graph. Scale bar = 100 μm, *p‐value < 0.05, **p‐value < 0.01.

To confirm the result based on a cancer cells**‐**line, we investigated the effect of Wnt signalling activators on human primary ameloblastoma cells, which were freshly isolated from excised ameloblastoma tissue (Figure 4). Similar with the result observed in AM**‐**1 cells, VPA increased cell size and decreased cell proliferation in a dose**‐**dependent manner (Figure 4A‐C). The VPA**‐**dose**‐**dependent increase in β**‐**catenin was also observed in the primary ameloblastoma cells (Figure S5A,B).

An antibody to sclerostin was observed to act as a bone anabolic agent by neutralizing antagonists of Wnt ligands.^23^ In our study, the sclerostin antibody (SOST antibody) showed similar effect with other chemical Wnt signalling activators; the SOST antibody increased cell size and decreased cell proliferation of primary ameloblastoma cells (Figure 4D‐F).

### 3.6 Effect of a Wnt signalling activator on suspension‐cultured AM‐1 cells

**FIGURE 5:**
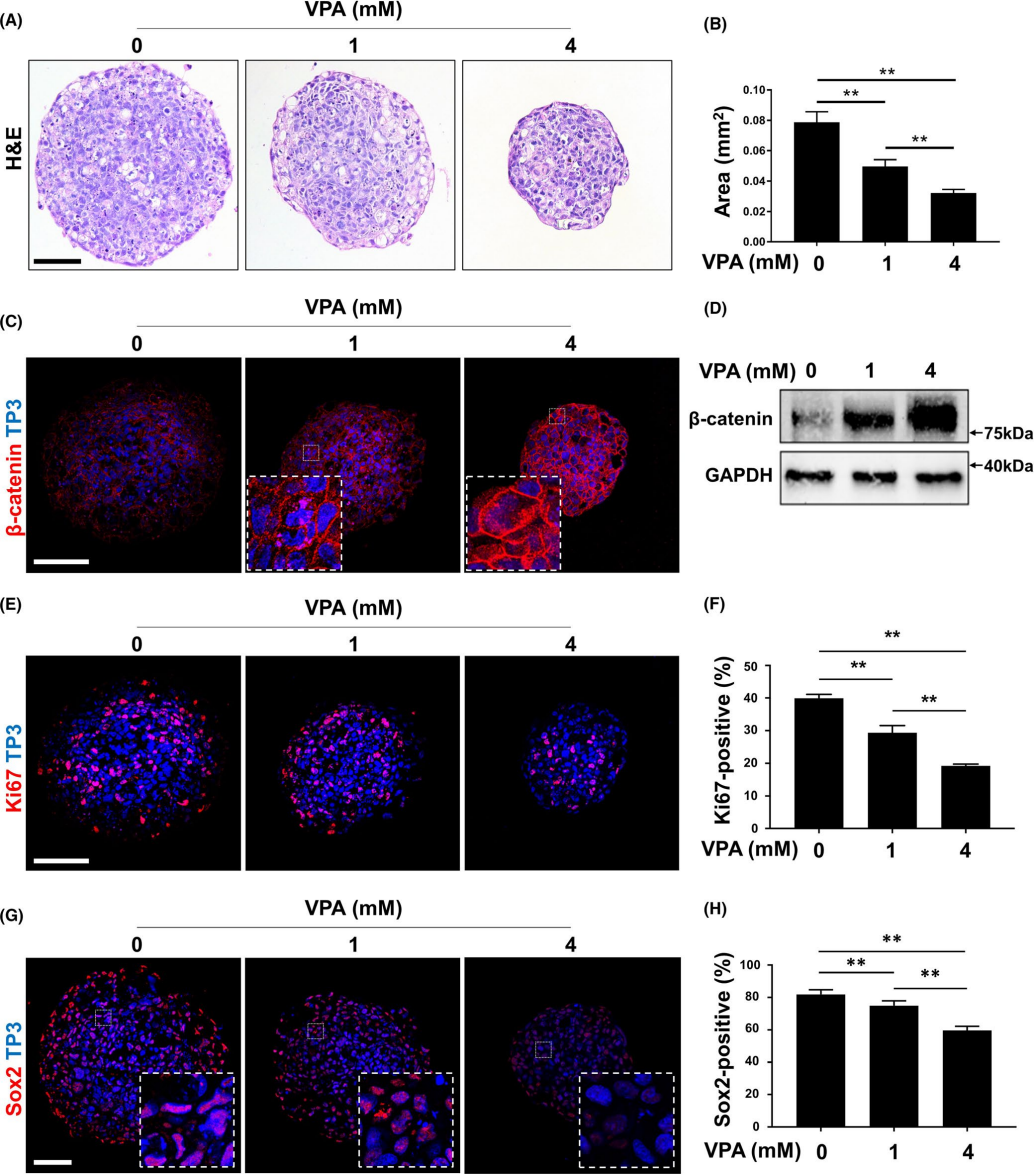
Suspended culture of AM**‐**1 cells and effects of VPA on the growth. A**‐**H, Two**‐**hundred**‐**thousand AM**‐**1 cells were plated on a well of a low**‐**attachment surface 6**‐**well cell culture plate and cultured for 7 d in DMEM with or without VPA. Sections of spheroids were subjected to H&E staining (A), immunohistochemistry using antibodies to β**‐**catenin (C, red), Ki67 (E, red) and Sox2 (G, red); and immunoblotting using antibodies to β**‐**catenin or GAPDH (D). Nuclei were visualized using (C, E and G, blue). Scale bar = 200 μm. The size of sectioned spheroids (B) and ratio of Ki67**‐**positive (F) or Sox2**‐**positive (H) nuclei were quantified. n = 50. **p **‐**value < 0.01. VPA, valproic acid, TP3, TO**‐**PRO**‐**3

To investigate the effect of Wnt signalling on the formation of AM**‐**1 spheroids, we activated Wnt signalling in the spheroids by treatment with VPA. The sectioned spheroids showed a dose**‐**dependent decrease in size (Figure 5A,B). Immunostaining and immunoblotting confirmed an increase in β**‐**catenin by VPA treatment of the spheroids (Figure 5C,D). With an increase in β**‐**catenin, the expression of a proliferation marker, Ki67, and a stem cell marker, Sox2, was decreased (Figure 5C‐H). However, cleaved Caspase 3, a marker of apoptosis, was not changed by VPA treatment (Figure S6).

**FIGURE 6:**
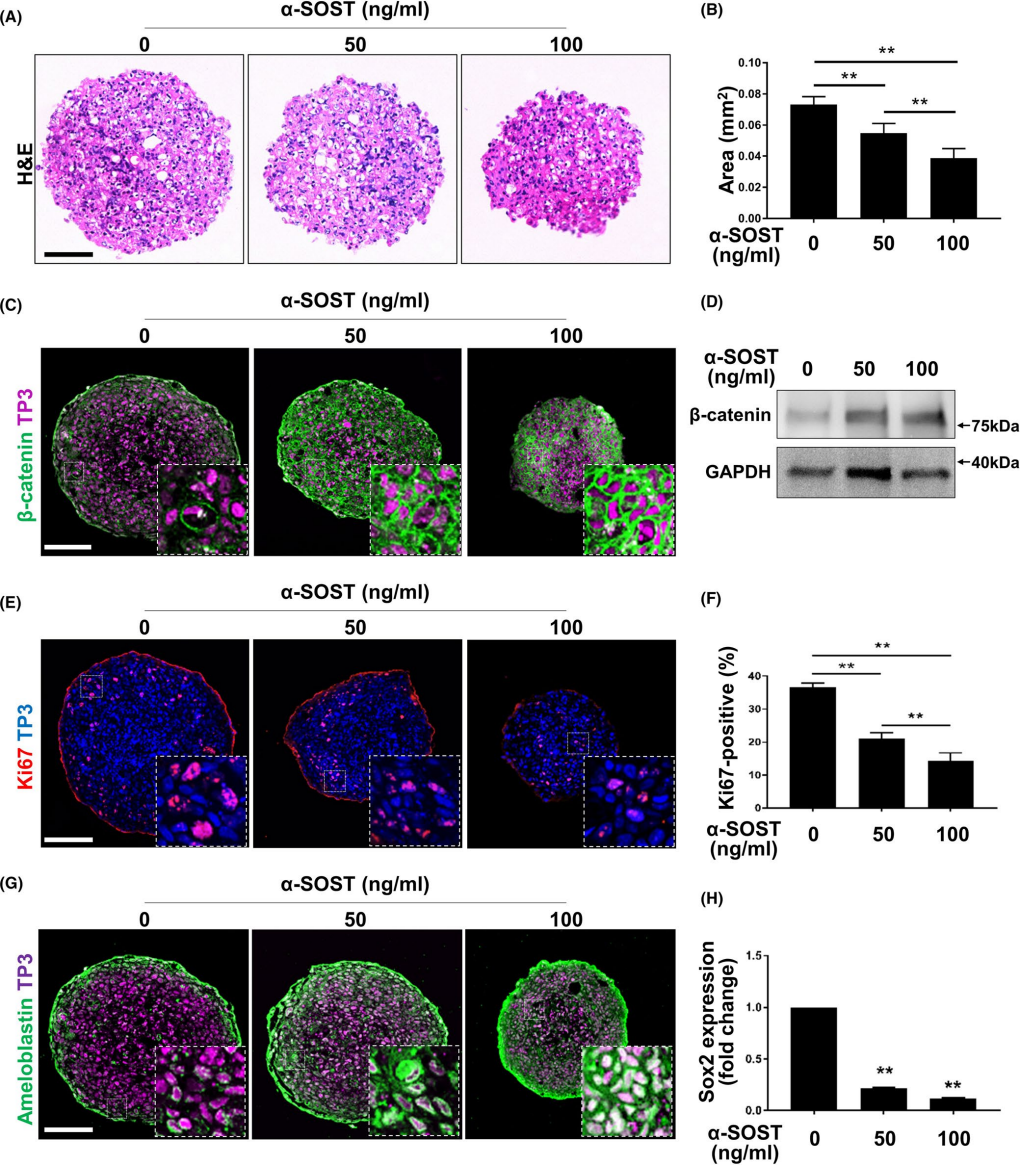
Suspended culture of AM**‐**1 cells and effects of sclerostin antibody on their growth. A**‐**H, Two**‐**hundred**‐**thousand AM**‐**1 cells were plated on a well of low**‐**attachment surface 6 well cell culture plate and cultured for 7 d in DMEM with or without sclerostin antibody (α**‐**SOST). Sections of spheroids were subjected to H&E staining (A); immunohistochemistry using antibodies to β**‐**catenin (C, green), Ki67 (E, red), and ameloblastin (G, green); and immunoblotting using antibodies to β**‐**catenin or anti**‐**GAPDH (D). Nuclei were visualized using TP3. Scale bar = 200 μm. The size of sectioned spheroids (B) and ratio of Ki67**‐**positive (F) or Sox2**‐**positive (H) nuclei were quantified. n = 50. **p **‐**value < 0.01. α**‐**SOST, anti**‐**SOST antibody, TP3, TO**‐**PRO**‐**3

The SOST antibody also reduced the size of AM**‐**1 spheroids (Figure 6A,B). The β**‐**catenin level was increased, and the number of Ki67**‐**positive cells decreased in the spheroids after treatment with the antibody (Figure 6C‐F). Notably, the expression of ameloblastin, a marker of ameloblast differentiation, was increased by the antibody (Figure 6G,H). However, no significant effect of the antibody was observed on the apoptosis of AM**‐**1 spheroids (Figure S7).

### 3.7 Effect of a Wnt signalling activator on 3D‐cultured AM‐1 cells in gels

**FIGURE 7:**
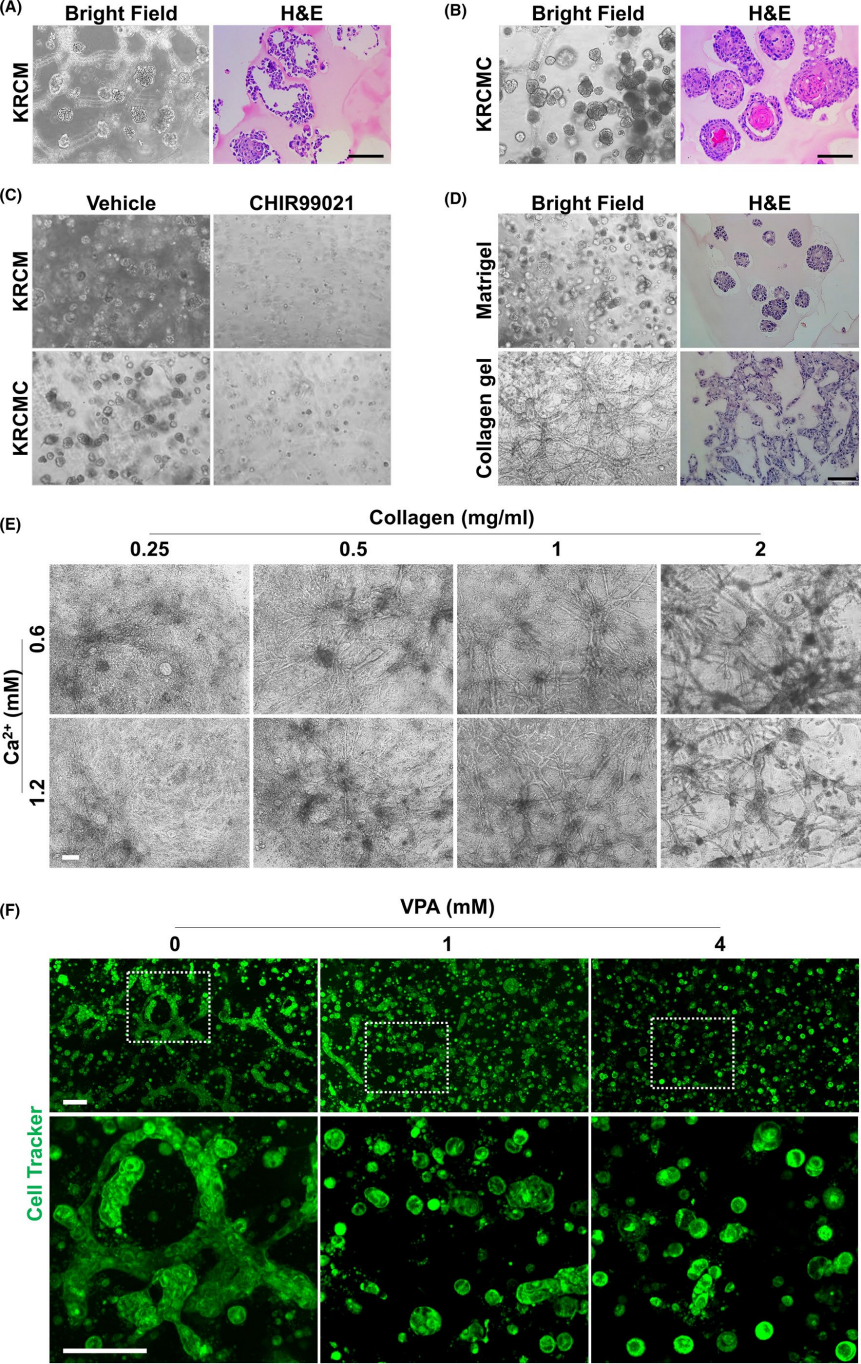
Three**‐**dimensional culture of AM**‐**1 cells and effects of Wnt activators on their growth. A**‐**F, AM**‐**1 cells embedded in Matrigel (A**‐**D) or collagen gel (1 mg/ml for D, 2 mg/ml for F, and E as indicated) were grown in keratinocyte media with (KRCMC, 0.6 mmol/L for a**‐**d, 1.2 mmol/L for F, and E as indicated) or without Ca^2+^ supplement (KRCM) for 14 days. VPA was added to the media at the indicated doses (F). Scale bar = 100 μm. VPA, valproic acid

Recent advances in 3D cell culture have enabled the development of more physiological in vitro models using cancer cells.^24^ First, we tried 3D culture of AM**‐**1 cells in Matrigel, a well**‐**defined soluble basement membrane used widely.^25^ Initially, we cultured AM**‐**1 cells in 3D Matrigel using a keratinocyte medium. The cells were observed to proliferate in Matrigel; however, they formed loosely connected cell masses (Figure 7A). When Ca^2+^ was supplied to the medium, AM**‐**1 cells adhered to each other to form tightly compacted cell spheroids (Figure 7B). The spheroids showed a complex structure – a basal cell**‐**like hematoxylinophilic outer region and keratinizing eosinophilic inner region were observed (Figure 7B). In both cases, exogenous activation of Wnt signalling using CHIR99021, a GSK3β inhibitor, suppressed proliferation and mass formation of the cells (Figure 7C). Next, to provide an environment more similar to an in vivo microenvironment, we cultured the cells in collagen gel. Interestingly, a network**‐**like growth pattern of AM**‐**1 cells was observed in the collagen gel (Figure 7D). In addition, the cells formed more prominent and thicker networks at higher concentrations of Ca^2+^ and collagen (Figure 7E). Wnt signalling activators, VPA and CHIR99021, suppressed growth of the cells in the collagen gel in a dose**‐**dependent manner (Figure 7F and Figure S8).

## 4 DISCUSSION

Radical surgery is the current mainstay of ameloblastoma treatment and includes an en bloc resection of 1‐2 cm of the bone adjacent to the ameloblastoma, which usually requires immediate bone reconstruction for speech and swallowing. This reconstruction is not necessary for conservative surgery, such as simple enucleation or curettage of the bone margin. However, the rate of recurrence after these operations can be as high as 60%‐90%.^5^ With such high recurrence rates, the characteristics of ameloblastoma, such as slow**‐** and long**‐**term proliferation, resistance to radiation and chemotherapy, and complex histological structures within the tumours, strongly imply the possible existence of CSCs in ameloblastoma. However, very few pathological studies have reported stem cell marker staining in tumour tissue,^22^, ^26^ and the characteristics and roles of CSCs in tumorigenesis remain to be elucidated. In this study, we characterized a putative CSC population in AM**‐**1 cells, a well**‐**established human ameloblastoma cell line. We observed that the small round cell population of AM**‐**1 cells was proliferative and expressed a stem cell marker. We also found that AM**‐**1 cells possessed spheroid**‐**forming capacity, which is an indicator for stemness of cells. In addition, the orthotopic graft of AM**‐**1 cells formed a mass that exhibited keratinization. These findings may serve to pave the way to study CSC populations of ameloblastoma in vitro.

**FIGURE 8:**
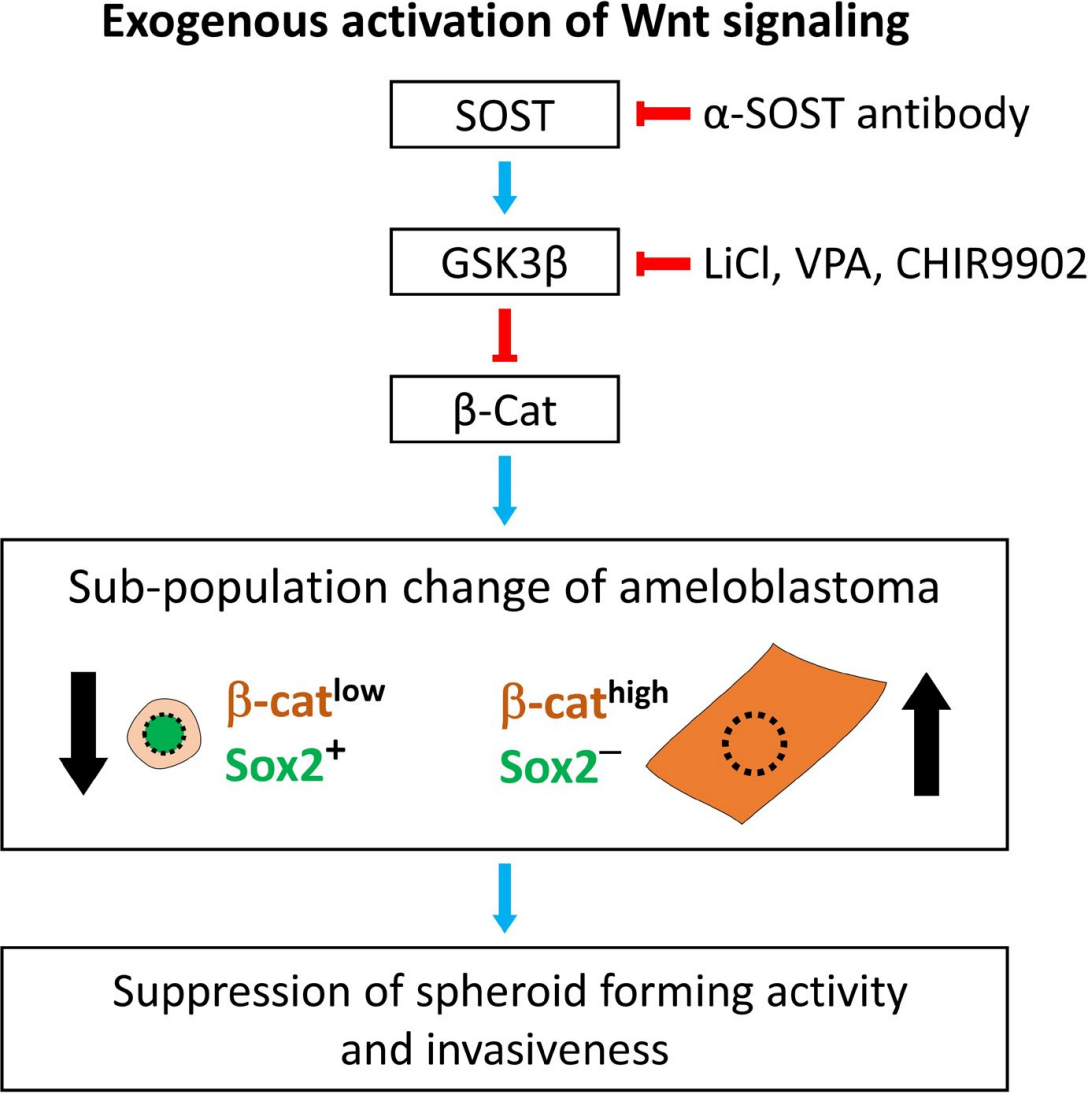
Schematic diagram for the effect of Wnt signalling activators on ameloblastoma. Exogenous activation of Wnt signalling using antibody (α**‐**SOST antibody) or chemicals (LiCl, VPA, or CHIR9902) increases β**‐**catenin (β**‐**cat) in ameloblastoma, leading to an increase in β**‐**cat^high^ and Sox2**^‐^** cells and decrease in β**‐**cat^low^ and Sox2^+^ cells, the putative cancer stem cells. This change of subpopulation in ameloblastoma results in the suppression of spheroid**‐**forming activity and invasiveness

Wnt signalling is an osteogenic signalling pathway that promotes bone generation by activating osteogenesis and inhibiting osteoclastogenesis.^27^ In tooth development, Wnt signalling is known to be activated in the late stage of development and facilitates ameloblast differentiation and movement.^19^ Expression of Sfrp, an antagonist of Wnt signalling, is observed in Sox2**‐**positive DESCs.^18^ Interestingly, AM**‐**1 cell line also expresses the Wnt antagonist Sfrp2,^16^ and osteogenic genes related to Wnt signalling are suppressed in this cell line.^17^ Here, we revealed that the Sox2**‐**positive population of the AM**‐**1 cell line showed low Wnt signalling activity. Exogenous activation of Wnt signalling by treatment with chemical Wnt activators (LiCl, VPA and CHIR9902) reduced the number of Sox2**‐**positive cells, spheroid**‐**forming capacity and invasiveness of AM**‐**1 cells into collagen gel (Figure 8). Recently, a sclerostin antibody was approved as a therapeutic agent against osteoporosis.^28^ The antibody activates Wnt signalling by neutralizing sclerostin, a Wnt antagonist secreted by mature osteocytes, to prevent excessive osteogenesis.^29^ A recent study revealed that treatment with the antibody stimulates mandibular bone formation.^23^ In our study, treatment with sclerostin antibody activated Wnt signalling and suppressed the formation of AM**‐**1 spheroids (Figure 8). The effect of Wnt signalling activators was clearly confirmed by human primary ameloblastoma cells. Taken together, we suggest that treatment with Wnt activators is a potential therapeutic option after resection of ameloblastoma, which promotes bone healing along with suppression of tumour recurrence. AM**‐**1 cells showed anchorage**‐**independent growth, which is a property of transformed cells.^30^ Proliferation of AM**‐**1 cells was observed in a suspension culture containing keratinocyte growth medium that had a low calcium concentration,^31^ as well as that containing DMEM. However, spheroid formation was only observed in suspension culture containing DMEM. The suspended AM**‐**1 cells in DMEM formed compact and round spheroids that were approximately 200 μm in diameter. By contrast, small and loosely connected cell masses were observed in the keratinocyte growth medium. A similar phenomenon was observed in 3D cultures using Matrigel and collagen gel. Calcium is known to be an important co**‐**factor of cell adhesion molecules that affects cell differentiation and migration.^32^ Further studies are required to reveal the role of calcium in spheroid**‐**forming activity of AM**‐**1 cells. Notably, AM**‐**1 cells showed network**‐**like growth in collagen gel, which is similar to the growth pattern of plexiform ameloblastoma. In addition, culture conditions should be further optimized to represent more characteristics, such as reverse polarization of cells from the basement membrane of ameloblastoma.

## 5 CONCLUSION

In conclusion, we identified putative CSC population in human immortalized and primary ameloblastoma cells. Furthermore, we showed that exogenous activation of Wnt signalling decreased the CSC population and suppressed their 2**‐** and 3**‐**dimensional growth of ameloblastoma cells. This finding suggests that the Wnt signalling activators could be developed as drugs suppressing CSCs in ameloblastoma, which could provide an alternative option to treat cancer without radical destructive surgery.

## CONFLICTS OF INTEREST

The authors declare that they have no competing interests.

## AUTHOR CONTRIBUTIONS

H**‐**Y. Kim, S. Li and D**‐**J. Lee performed the experiments. H**‐**Y. Kim, J. H. Park, Y**‐**S. Jung and H**‐**S. Jung designed the experiments and analysed the data. H**‐**Y. Kim, T. Muramatsu, H. Harada, Y**‐**S. Jung and H**‐**S. Jung wrote the manuscript.

## Supporting information

Fig S1‐8

## Data Availability

All data generated or analysed during this study are included in this published article.

## References

[1] van Niekerk G , Davids LM , Hattingh SM , Engelbrecht AM . Cancer stem cells: a product of clonal evolution? Int J Cancer. 2017;140(5):993‐999. 27676693 10.1002/ijc.30448

[2] Carnero A , Garcia‐Mayea Y , Mir C , Lorente J , Rubio IT , LLeonart ME . The cancer stem**‐**cell signaling network and resistance to therapy. Cancer Treat Rev. 2016;49:25‐36. 27434881 10.1016/j.ctrv.2016.07.001

[3] El**‐**Naggar AK , Chan JKC , Takata T , Grandis JR , Slootweg PJ . The fourth edition of the head and neck world health organization blue book: editors' perspectives. Human Pathology. 2017;66:10‐12. 28583885 10.1016/j.humpath.2017.05.014

[4] Sham E , Leong J , Maher R , Schenberg M , Leung M , Mansour AK . Mandibular ameloblastoma: clinical experience and literature review. ANZ J Surg. 2009;79(10):739‐744. 19878171 10.1111/j.1445-2197.2009.05061.x

[5] McClary AC , West RB , McClary AC , et al. Ameloblastoma: a clinical review and trends in management. Eur Arch Otorhinolaryngol. 2016;273(7):1649‐1661. 25926124 10.1007/s00405-015-3631-8

[6] Sweeney RT , McClary AC , Myers BR , et al. Identification of recurrent SMO and BRAF mutations in ameloblastomas. Nat Genet. 2014;46(7):722‐725. 24859340 10.1038/ng.2986 PMC4418232

[7] Harada H , Mitsuyasu T , Nakamura N , et al. Establishment of ameloblastoma cell line, AM**‐**1. J Oral Pathol Med. 1998;27(5):207‐212. 9682983 10.1111/j.1600-0714.1998.tb01943.x

[8] Sandra F , Hendarmin L , Kukita T , Nakao Y , Nakamura N , Nakamura S . Ameloblastoma induces osteoclastogenesis: a possible role of ameloblastoma in expanding in the bone. Oral Oncol. 2005;41(6):637‐644. 15935726 10.1016/j.oraloncology.2005.02.008

[9] Takebe Y , Tsujigiwa H , Katase N , et al. Parenchyma‐stromal interactions induce fibrosis by secreting CCN2 and promote osteoclastogenesis by stimulating RANKL and CD68 through activated TGF‐beta/BMP4 in ameloblastoma. J Oral Pathol Med. 2016;46(1):67‐75. 27327904 10.1111/jop.12467

[10] Yoshimoto S , Morita H , Matsubara R , et al. Surface vacuolar ATPase in ameloblastoma contributes to tumor invasion of the jaw bone. Int J Oncol. 2016;48(3):1258‐1270. 26794206 10.3892/ijo.2016.3350

[11] Sandra F , Harada H , Nakamura N , Ohishi M . Midkine induced growth of ameloblastoma through MAPK and Akt pathways. Oral Oncol. 2004;40(3):274‐280. 14747058 10.1016/j.oraloncology.2003.08.011

[12] Hendarmin L , Sandra F , Nakao Y , Ohishi M , Nakamura N . TNFalpha played a role in induction of Akt and MAPK signals in ameloblastoma. Oral Oncol. 2005;41(4):375‐382. 15792609 10.1016/j.oraloncology.2004.09.014

[13] Sandra F , Hendarmin L , Nakao Y , Nakamura N , Nakamura S . Inhibition of Akt and MAPK pathways elevated potential of TNFalpha in inducing apoptosis in ameloblastoma. Oral Oncol. 2006;42(1):39‐45. 16140562 10.1016/j.oraloncology.2005.04.011

[14] Kanda S , Mitsuyasu T , Nakao Y , et al. Anti‐apoptotic role of the sonic hedgehog signaling pathway in the proliferation of ameloblastoma. Int J Oncol. 2013;43(3):695‐702. 23835807 10.3892/ijo.2013.2010 PMC3787891

[15] Nakao Y , Mitsuyasu T , Kawano S , Nakamura N , Kanda S , Nakamura S . Fibroblast growth factors 7 and 10 are involved in ameloblastoma proliferation via the mitogen‐activated protein kinase pathway. Int J Oncol. 2013;43(5):1377‐1384. 24002438 10.3892/ijo.2013.2081 PMC3823399

[16] Sathi GA , Inoue M , Harada H , et al. Secreted frizzled related protein (sFRP)‐2 inhibits bone formation and promotes cell proliferation in ameloblastoma. Oral Oncol. 2009;45(10):856‐860. 19362047 10.1016/j.oraloncology.2009.02.001

[17] Sathi GA , Tsujigiwa H , Ito S , et al. Osteogenic genes related to the canonic WNT pathway are down‐regulated in ameloblastoma. Oral Surg Oral Med Oral Pathol Oral Radiol. 2012;114(6):771‐777. 23159115 10.1016/j.oooo.2012.08.453

[18] Juuri E , Saito K , Ahtiainen L , et al. Sox2+ stem cells contribute to all epithelial lineages of the tooth via Sfrp5+ progenitors. Dev Cell. 2012;23(2):317‐328. 22819339 10.1016/j.devcel.2012.05.012 PMC3690347

[19] Guan X , Xu M , Millar SE , Bartlett JD . Beta‐catenin is essential for ameloblast movement during enamel development. Eur J Oral Sci. 2016;124(3):221‐227. 26957367 10.1111/eos.12261

[20] Juuri E , Jussila M , Seidel K , et al. Sox2 marks epithelial competence to generate teeth in mammals and reptiles. Development. 2013;140(7):1424‐1432. 23462476 10.1242/dev.089599 PMC3596986

[21] Lee MJ , Kim EJ , Otsu K , Harada H , Jung HS . Sox2 contributes to tooth development via Wnt signaling. Cell Tissue Res. 2016;365(1):77‐84. 26846112 10.1007/s00441-016-2363-4

[22] Chang JY , Wang C , Jin C , et al. Self‐renewal and multilineage differentiation of mouse dental epithelial stem cells. Stem Cell Res. 2013;11(3):990‐1002. 23906788 10.1016/j.scr.2013.06.008 PMC3952636

[23] Tamplen M , Fowler T , Markey J , Knott PD , Suva LJ , Alliston T . Treatment with anti‐Sclerostin antibody to stimulate mandibular bone formation. Head Neck. 2018;40(7):1453‐1460. 29522281 10.1002/hed.25128 PMC6037571

[24] Nath S , Devi GR . Three‐dimensional culture systems in cancer research: focus on tumor spheroid model. Pharmacol Ther. 2016;163:94‐108. 27063403 10.1016/j.pharmthera.2016.03.013 PMC4961208

[25] Benton G , Arnaoutova I , George J , Kleinman HK , Koblinski J . Matrigel: from discovery and ECM mimicry to assays and models for cancer research. Adv Drug Deliv Rev. 2014;79‐80:3‐18. 10.1016/j.addr.2014.06.005 24997339

[26] Kumamoto H , Ohki K . Detection of CD133, Bmi‐1, and ABCG2 in ameloblastic tumors. J Oral Pathol Med. 2010;39(1):87‐93. 19659474 10.1111/j.1600-0714.2009.00807.x

[27] Monroe DG , McGee‐Lawrence ME , Oursler MJ , Westendorf JJ . Update on Wnt signaling in bone cell biology and bone disease. Gene. 2012;492(1):1‐18. 22079544 10.1016/j.gene.2011.10.044 PMC3392173

[28] Mullard A . FDA approves first‐in‐class osteoporosis drug. Nat Rev Drug Discov. 2019;18(6):411. 10.1038/d41573-019-00083-y 31160772

[29] McClung MR . Sclerostin antibodies in osteoporosis: latest evidence and therapeutic potential. Ther Adv Musculoskelet Dis. 2017;9(10):263‐270. 28974988 10.1177/1759720X17726744 PMC5613857

[30] Ju SY , Chiou SH , Su Y . Maintenance of the stemness in CD44(+) HCT‐15 and HCT‐116 human colon cancer cells requires miR‐203 suppression. Stem Cell Res. 2014;12(1):86‐100. 24145190 10.1016/j.scr.2013.09.011

[31] Pillai S , Bikle DD , Hincenbergs M , Elias PM . Biochemical and morphological characterization of growth and differentiation of normal human neonatal keratinocytes in a serum‐free medium. J Cell Physiol. 1988;134(2):229‐237. 2450102 10.1002/jcp.1041340208

[32] Tharmalingam S , Hampson DR . The calcium‐sensing receptor and integrins in cellular differentiation and migration. Front Physiol. 2016;7:190. 27303307 10.3389/fphys.2016.00190 PMC4880553

