# Supplementary material for: Activation of Wnt signalling reduces the population of cancer stem cells in ameloblastoma
Source: Cell Prolif. 2021 Jun 6;54(7):e13073. doi: 10.1111/cpr.13073 (PMC8249789; doi:10.1111/cpr.13073)
Supplement: Supplementary file 1 — Fig S1‐8 [file CPR-54-e13073-s001.docx]

**Activation of Wnt signalling reduces the population of cancer stem cells in ameloblastoma**

Hyun-Yi Kim, Shujin Li, Dong-Joon Lee, Jin Hoo Park, Takashi Muramatsu, Hidemitsu Harada, Young-Soo Jung and Han-Sung Jung

**
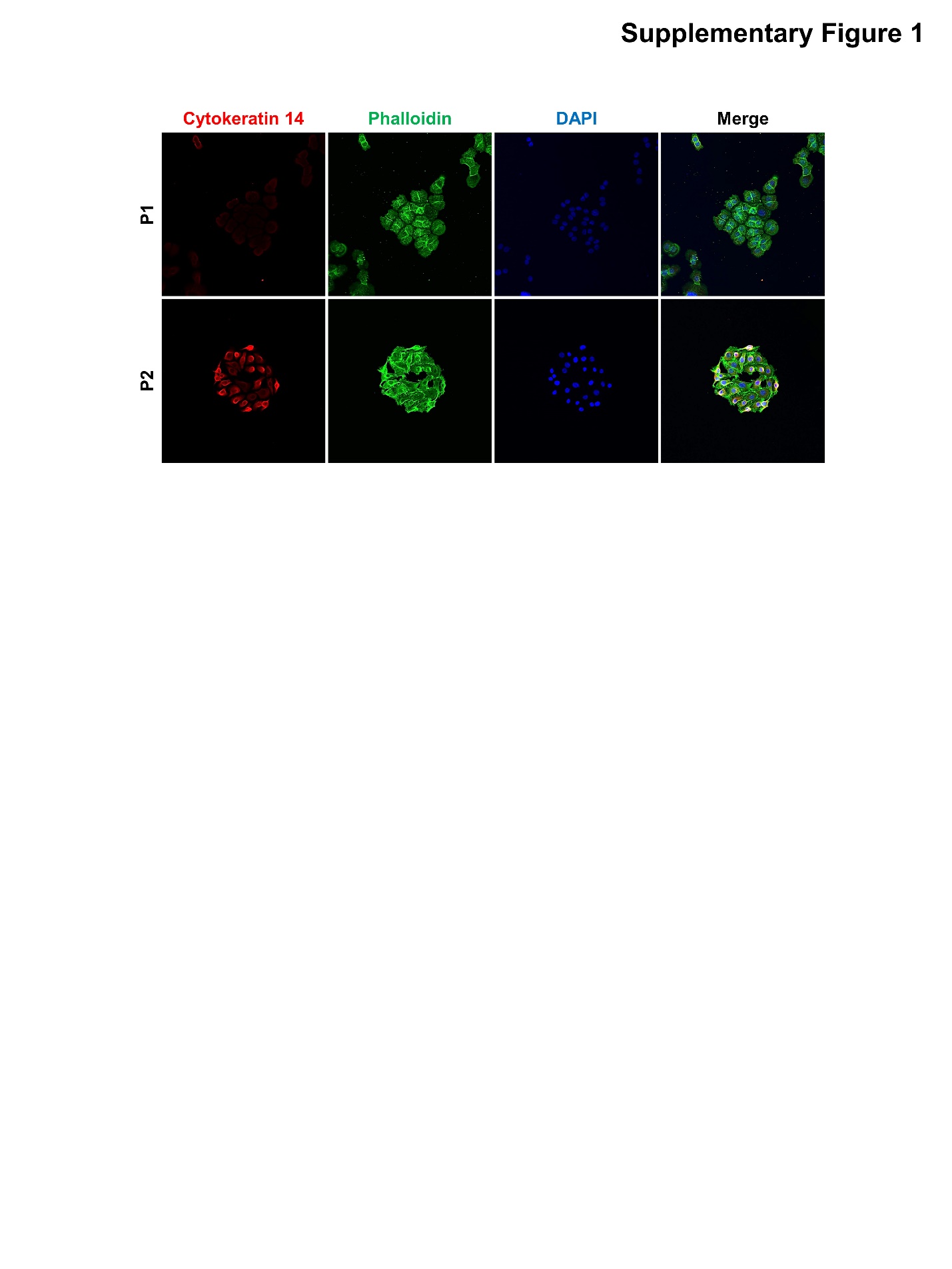
**

**Supplementary Figure 1** AM-1 harbours a cancer stem cell-like population. AM-1 cells were sorted into two groups (P1 and P2) based on FSC and SSC and cultured for 1 days. The cells were subjected to ICC using anti-Cytokeratin 14. The cytoskeleton and nucleus were visualized using fluorophore-conjugated Phalloidin (green) and DAPI (blue), respectively.
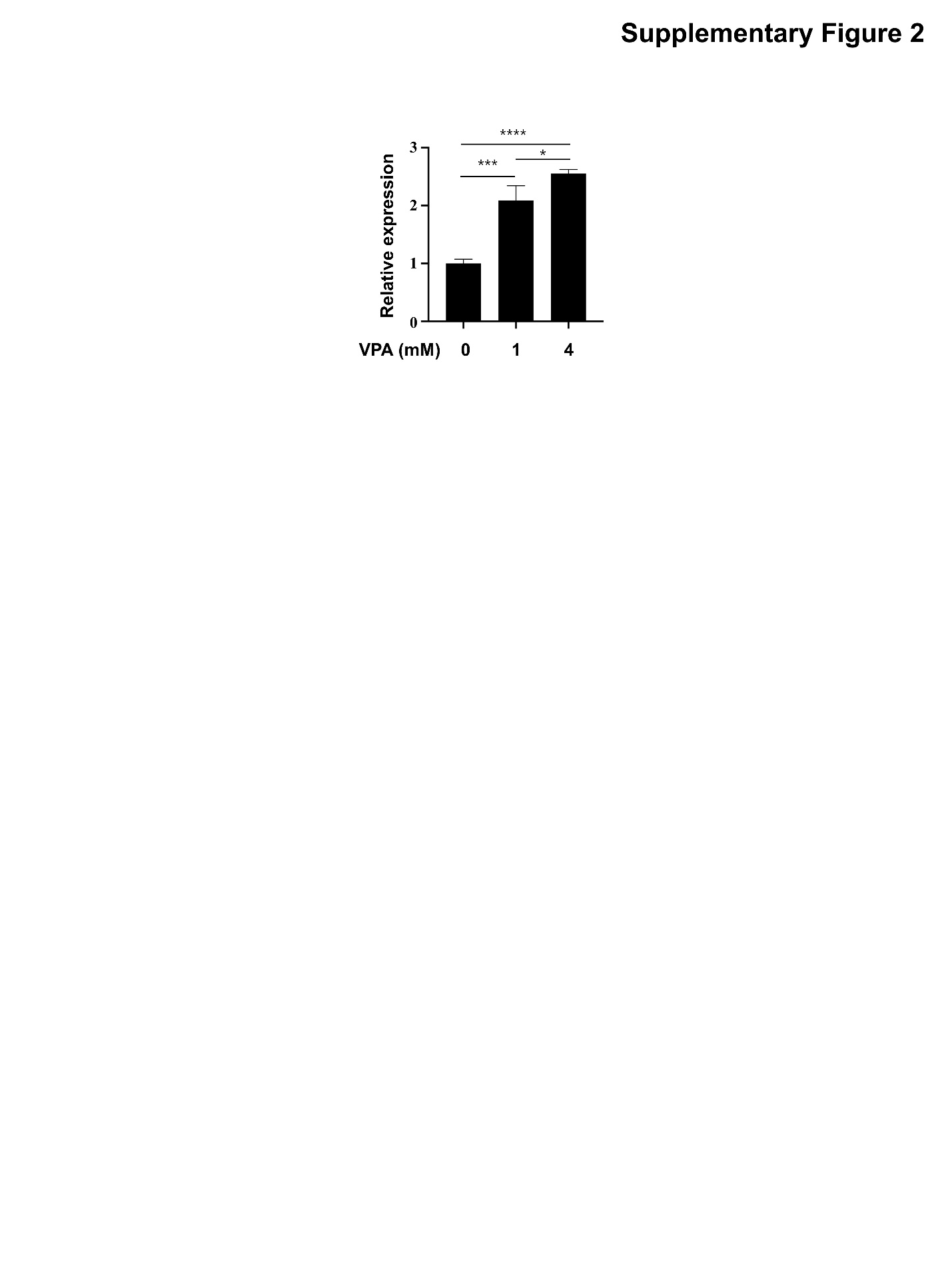


**Supplementary Figure 2** Total RNA of AM-1 cells cultured with or without valproic acid (VPA) were extracted and subjected to real-time PCR analyses using primer sets of *Axin2*. n = 3.

**
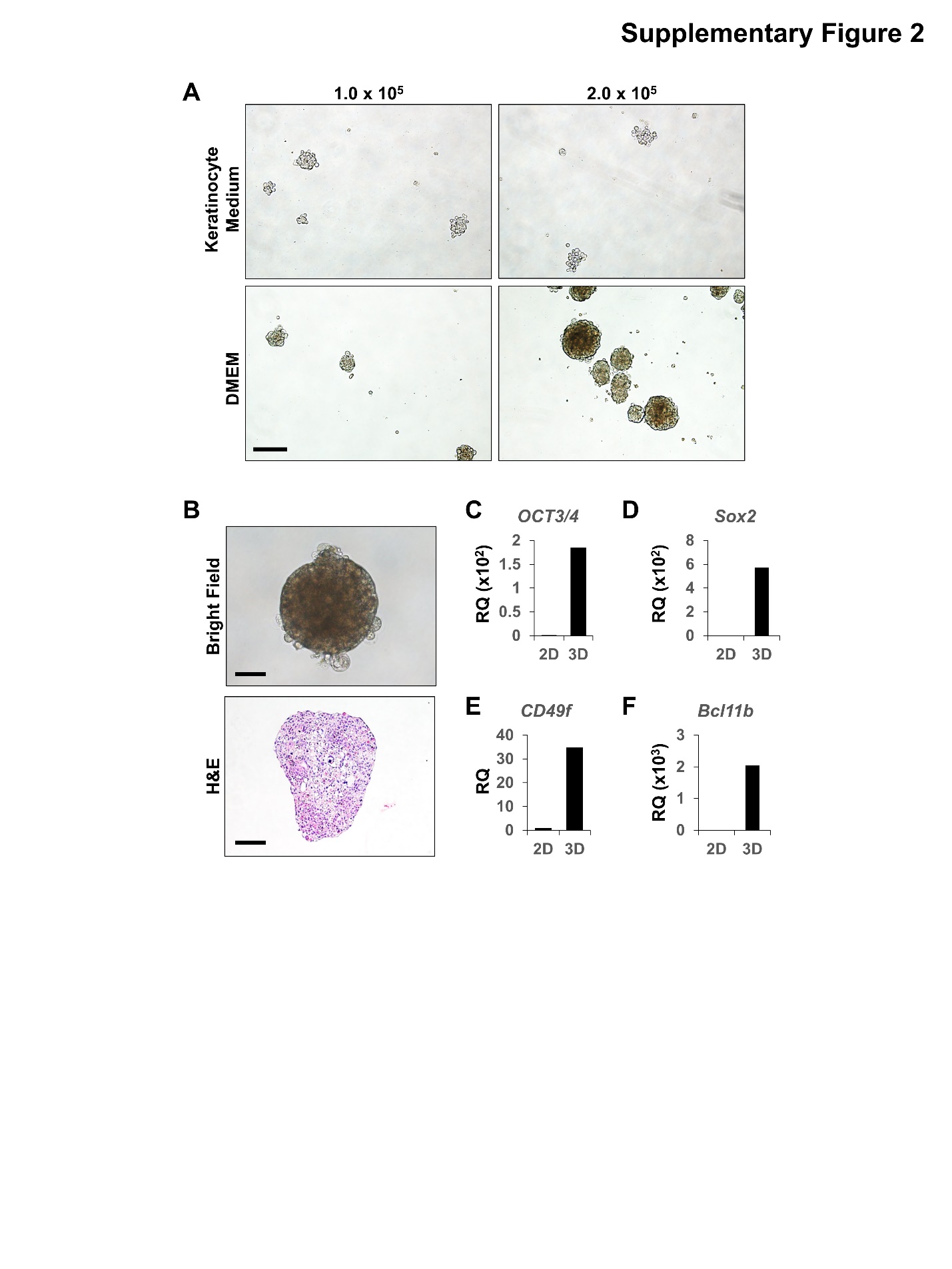
**

**Supplementary Figure 3** AM-1 shows spheroid forming activity. A, Indicated numbers of AM-1 cells were plated on a well of low attachment surface 6-well cell culture plates and cultured for 7 days in Keratinocyte growth medium or DMEM. Bright field images of a spheroids are displayed. Scale bar = 100 μm. B, Two-hundred-thousand AM-1 cells were plated on a well of low attachment surface 6-well cell culture plates and cultured for 7 days in DMEM. A bright field image of a spheroids (B, upper panel). Sections of spheroids were subjected into H&E staining (B, lower panel). Scale bar = 100 μm. C-F, Total RNA of AM-1 cells cultured in conventional 2-dimensional (2D) or 3-dimensional (3D) culture method were extracted and subjected to real-time PCR analyses using primer sets of *OCT3/4* (C), *Sox2* (D), *CD49f* (E), and *Bcl11b* (F). n = 3. RQ, relative quantity.

**
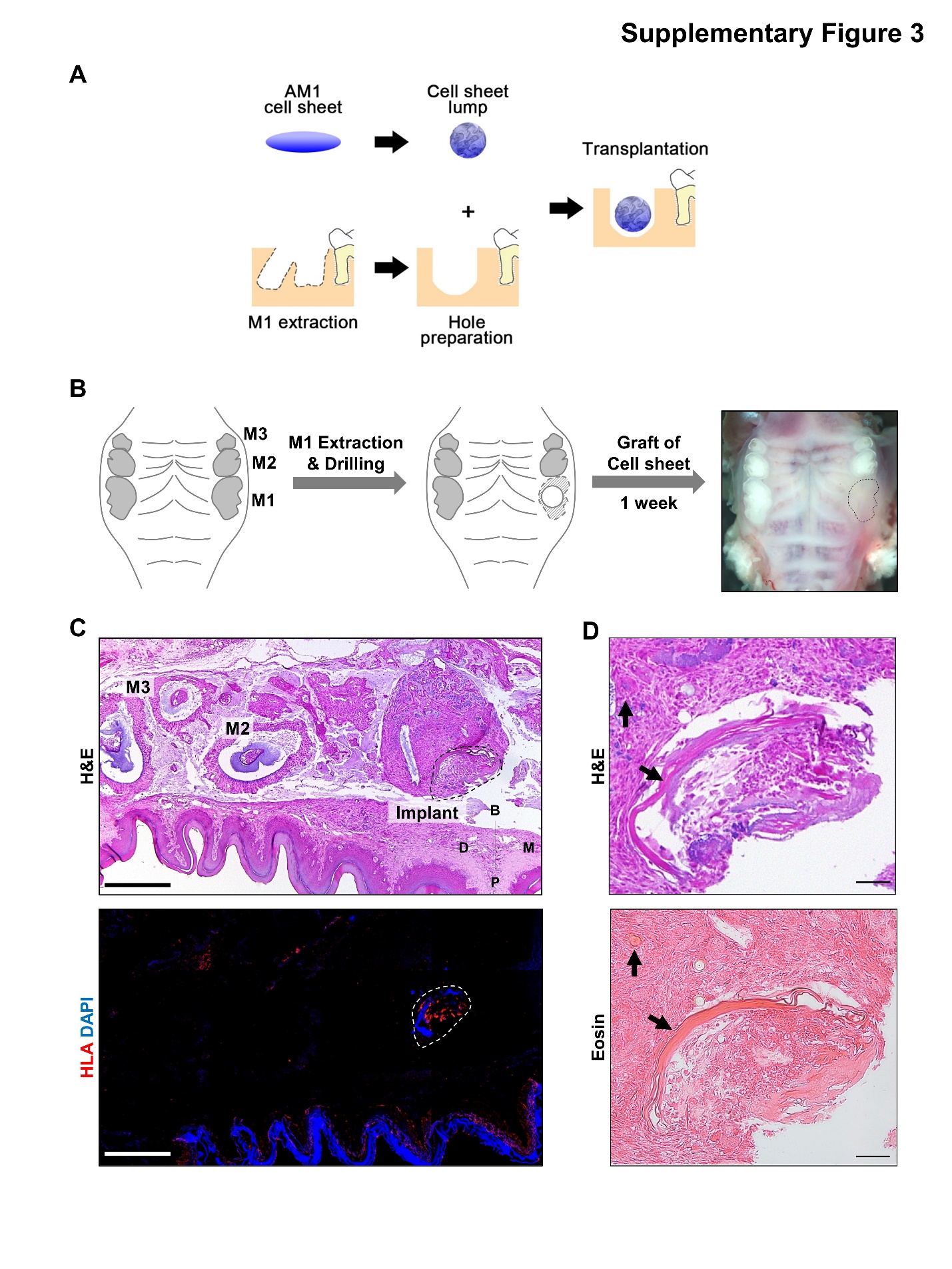
**

**Supplementary Figure 4** Schematic diagram of the orthotopic grafts of the AM-1 cell sheet into the maxillary of mouse. A, An AM-1 cell sheet lump was implanted into a hole prepared at the extraction site of the maxillary first molar of 8-week-old BALB/c nude mice. B, Schematic diagram of the orthotopic grafts of the AM-1 cell sheet into the maxillary first molar of an 8-week-old BALB/c nude mouse. C-D, Sections of maxillary tissue were subjected into H&E staining (C and D, upper panels), eosin staining (D, lower panel), or immunohistochemistry using anti human leukemia antigen (HLA) antibody (C, lower panel, red). The nucleus was visualized using DAPI (C, lower panel, blue). Scale bar c = 200 μm, d = 100 μm. M1, first molar. M2, second molar. M3, third molar.

**
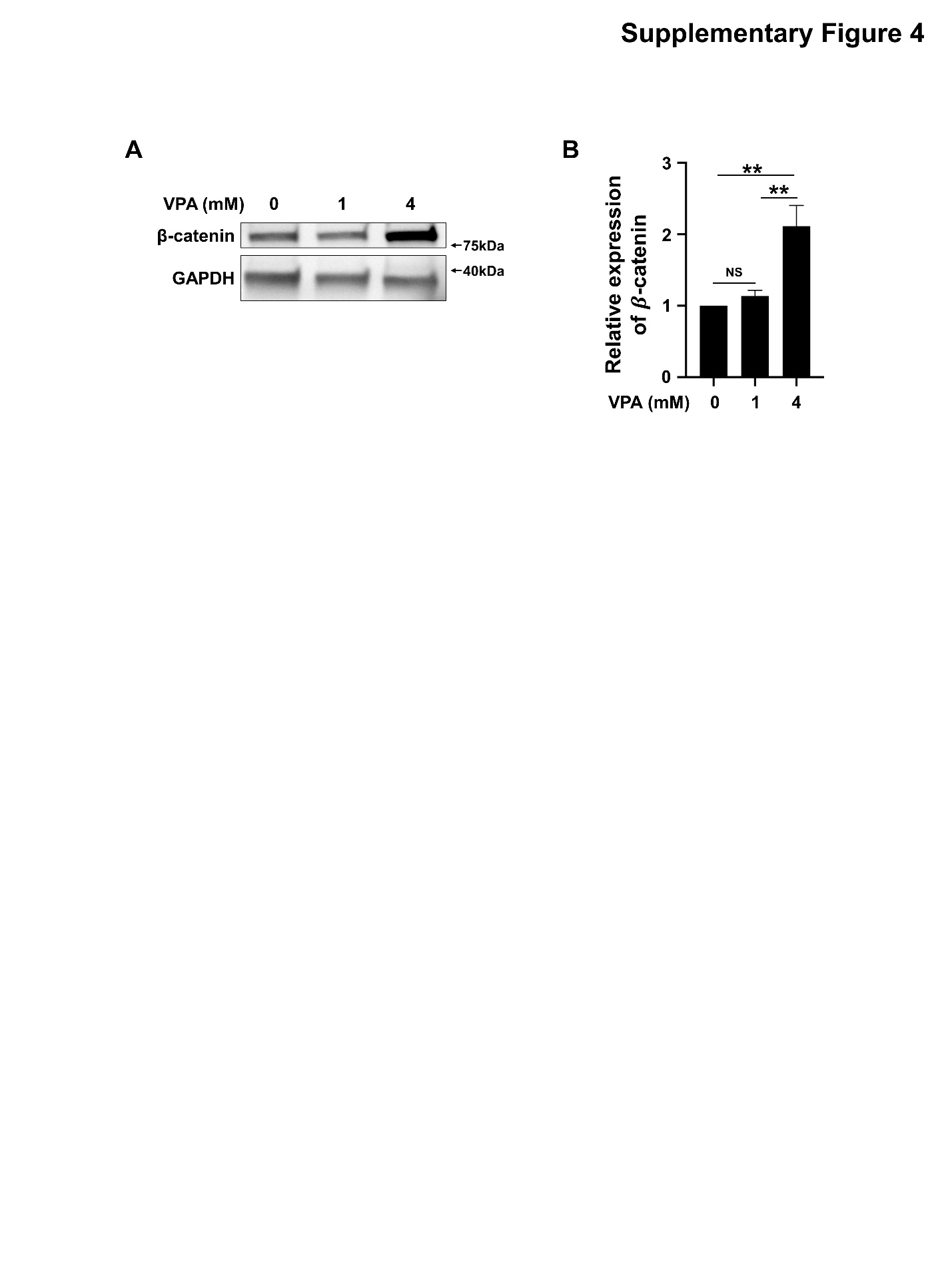
**

**Supplementary Figure 5** Treatment with VPA increased expression of β-catenin in human primary ameloblastoma cells. A-B, Human primary ameloblastoma cells were cultured for 24 h with the indicated dose of VPA. The cells were subjected to immunoblotting using antibodies to β-catenin or GAPDH (A) and the band intensity was quantified (B). **p-value<0.01.

**
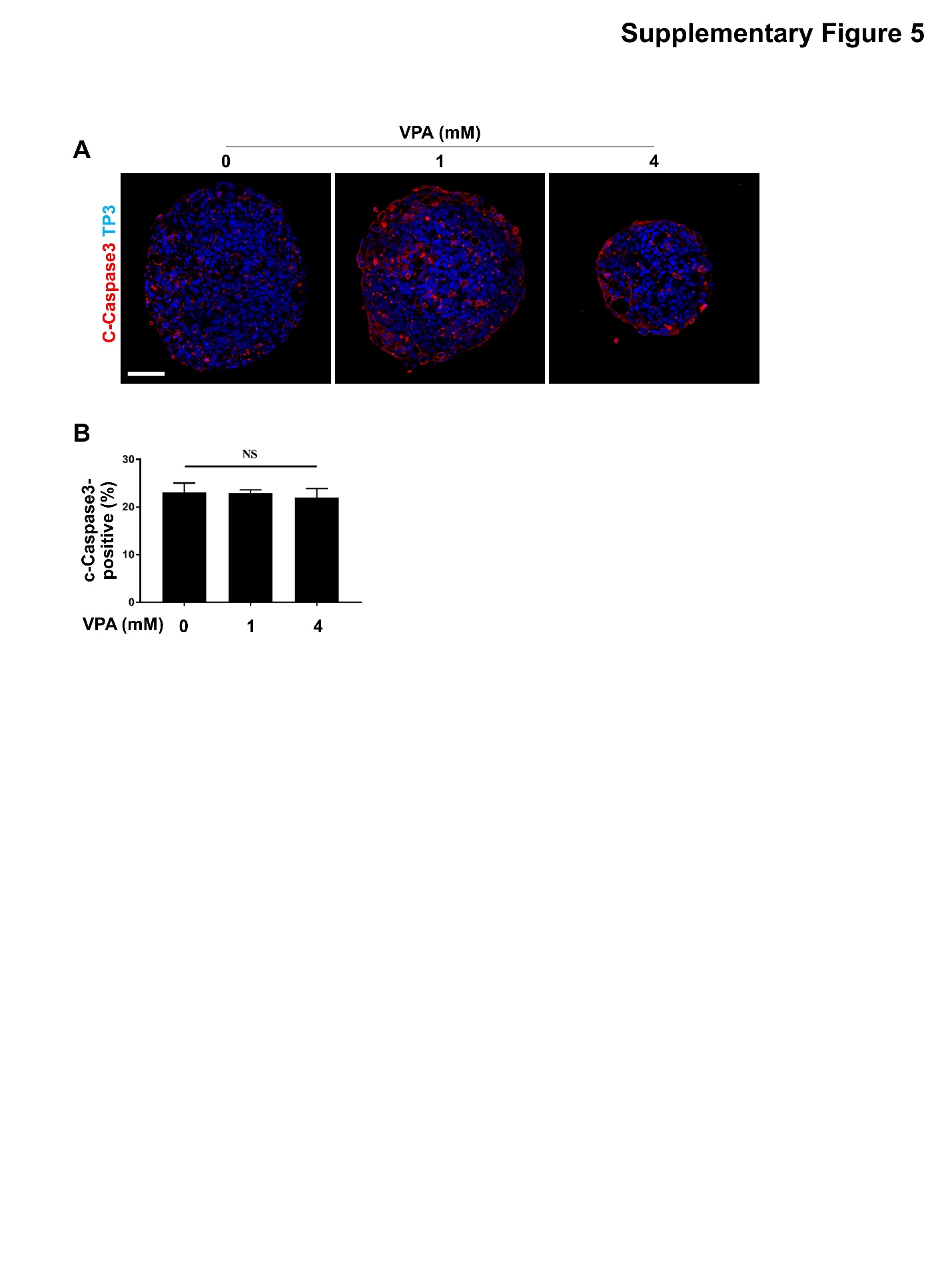
**

**Supplementary Figure 6** Expression of cleaved Caspase3 in VPA-treated AM-1 spheroid. A-B, Two-hundred-thousand AM-1 cells were plated on a well of low attachment surface 6-well cell culture plates and cultured for 7 days in DMEM with or without VPA. Sections of spheroid were subjected into immunohistochemistry using anti-cleaved Caspase3 (c-Caspase3, red) antibody (a, scale bar = 100 μm). The nucleus was visualized using TO-PRO-3 (TP3, A, blue). The c-Caspase3-positive area of spheroids was measured and displayed (B). n = 50. NS = not significant. VPA, valproic acid.

**
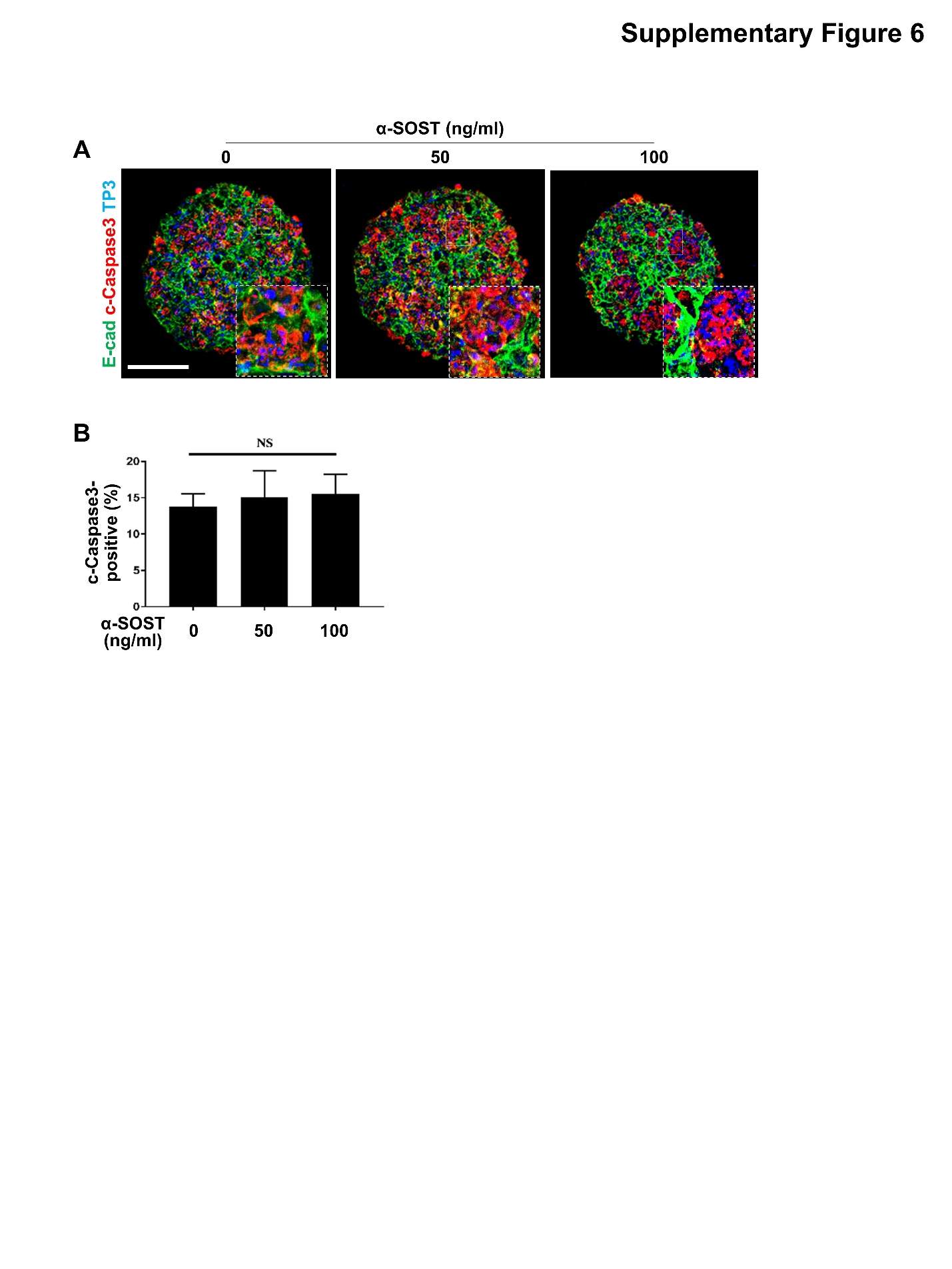
**

**Supplementary Figure 7** Expression of cleaved Caspase3 in anti-Sclerostin antibody-treated AM-1 spheroid. A-B, Two-hundred-thousand AM-1 cells were plated on a well of low attachment surface 6-well cell culture plates and cultured for 7 days in DMEM with or without anti-Sclerostin antibody. Sections of spheroid were subjected into immunohistochemistry using anti-cleaved Caspase3 (c-Caspase3, red) and E-cadherin (E-cad, green) antibodies (a, scale bar = 100 μm). The nucleus was visualized using TO-PRO-3 (TP3, A, blue). The c-Caspase3-positive area of spheroids was measured and displayed (B). n = 50. NS = not significant. α-SOST, anti-SOST antibody.

**
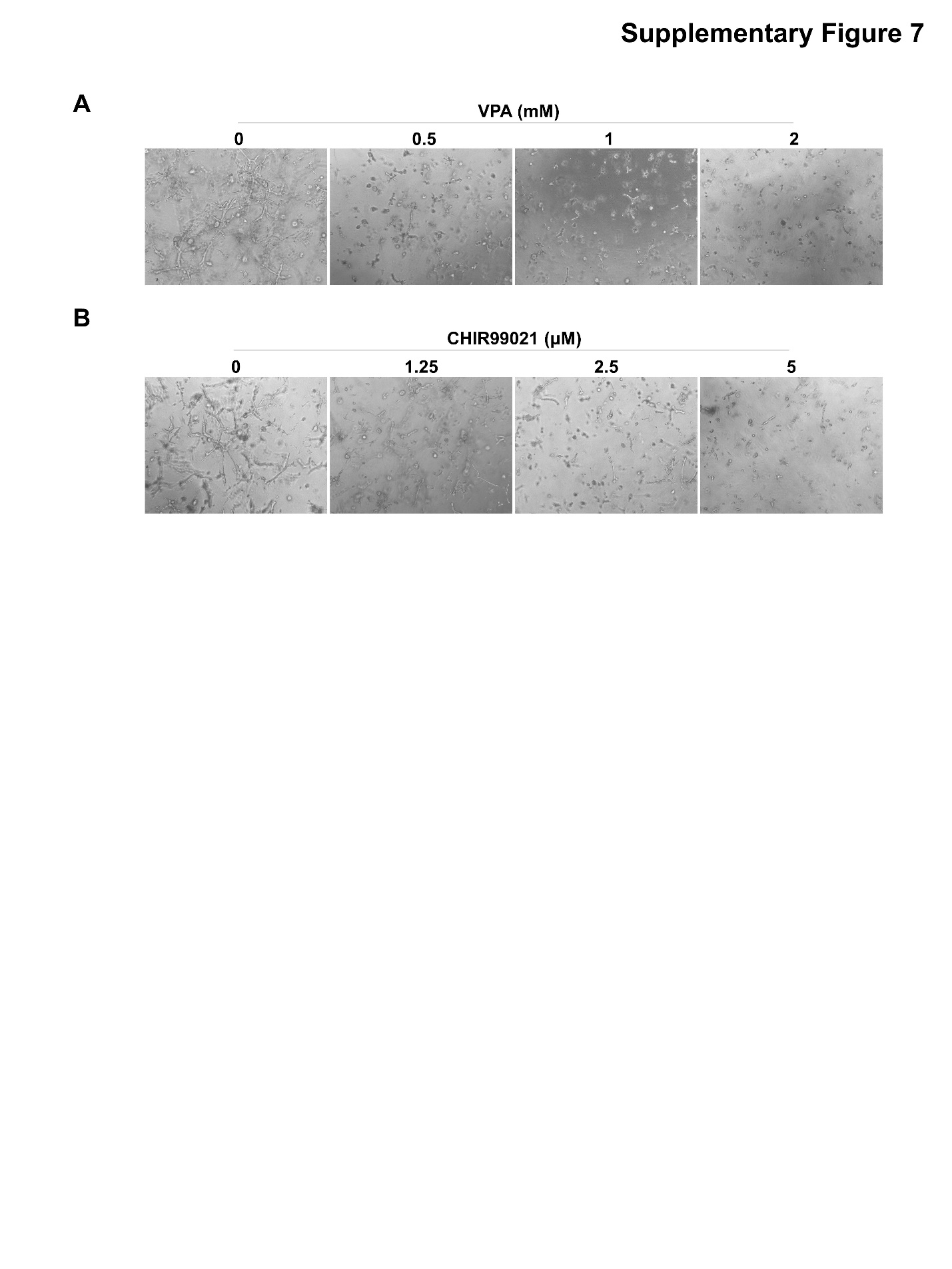
**

**Supplementary Figure 8** Three-dimensional culture of AM-1 cells and effects of Wnt activators on the growth. A-B, AM-1 cells embedded in collagen gel (2 mg/ml) were grown in Keratinocyte media with Ca^2+^ supplement (1.2 mM) for 14 days. VPA or CHIR99021 was added in media as indicated doses.
